# Inferring modules from human protein interactome classes

**DOI:** 10.1186/1752-0509-4-102

**Published:** 2010-07-23

**Authors:** Elisabetta Marras, Antonella Travaglione, Gautam Chaurasia, Matthias Futschik, Enrico Capobianco

**Affiliations:** 1 CRS4 Bioinformatics Laboratory - Technology Park of Sardinia, Pula (Cagliari), Sardinia - Italy; 2 Institute for Theoretical Biology - Humboldt University - Berlin, Germany; 3 Max-Delbrück Center for Molecular Medicine - Berlin, Germany; 4 Centre of Molecular and Structural Biomedicine - Universidade do Algarve - Faro, Portugal

## Abstract

**Background:**

The integration of protein-protein interaction networks derived from high-throughput screening approaches and complementary sources is a key topic in systems biology. Although integration of protein interaction data is conventionally performed, the effects of this procedure on the result of network analyses has not been examined yet. In particular, in order to optimize the fusion of heterogeneous interaction datasets, it is crucial to consider not only their degree of coverage and accuracy, but also their mutual dependencies and additional salient features.

**Results:**

We examined this issue based on the analysis of modules detected by network clustering methods applied to both integrated and individual (disaggregated) data sources, which we call interactome classes. Due to class diversity, we deal with variable dependencies of data features arising from structural specificities and biases, but also from possible overlaps. Since highly connected regions of the human interactome may point to potential protein complexes, we have focused on the concept of modularity, and elucidated the detection power of module extraction algorithms by independent validations based on GO, MIPS and KEGG. From the combination of protein interactions with gene expressions, a confidence scoring scheme has been proposed before proceeding via GO with further classification in permanent and transient modules.

**Conclusions:**

Disaggregated interactomes are shown to be informative for inferring modularity, thus contributing to perform an effective integrative analysis. Validation of the extracted modules by multiple annotation allows for the assessment of confidence measures assigned to the modules in a protein pathway context. Notably, the proposed multilayer confidence scheme can be used for network calibration by enabling a transition from unweighted to weighted interactomes based on biological evidence.

## Background

Networks are complex structures endowed with both statistical and topological properties ([1,2]). Biological networks, and protein-protein interaction networks (PPIN) in particular, require both theory and algorithms to describe complex mechanisms and relationships. Ideally, these networks can be assumed to represent snapshots depicted by connectivity maps observed at particular times. Through a sequence of such maps, we could verify how the network connectivity changes over time, and thus conceive a topological model for interpreting the dynamics and conducting inference by the built-in predictive power.

When time changes cannot be monitored, the available static pictures limit the potential for global interactome analysis. Despite this limitation, there has been a great interest in analyzing topological features of networks in order to cluster proteins into groups, assign functions to uncharacterized proteins, study their similarities, and establish reliability of the interactions. However, due to the impact of relatively low ratios between true and false positives and negatives, it is hard to accomplish those tasks before filtering interactome signals from noise.

Notably, comparative method evaluations ([3,4]) have been proposed to extract clusters of densely connected proteins which might indicate protein complexes or functional modules. The meaning of these two entities is distinct in biological terms (see [5] and [6] for an extended discussion). We refer to a protein complex (e.g. transcription factors, histones, polymerases, etc.) as a molecular machine consisting of multiple proteins (and possibly nucleic acids and other molecules) that bind at the same place and time. In contrast, a functional module (e.g. signaling pathways) represents a set of proteins (and other molecules) that controlor perform a particular cellular function but not necessarily at the same time and place, and thus may not form a macromolecular complex.

However, it is often hard to distinguish between these two structures by relying only on PPIN, as in general the analyzed protein interactions do not have temporal and spatial information. Nevertheless, since PPIN represent undirected binary or weighted graphs, several graph-based inference approaches have been successfully employed to detect modularity. The majority of such approaches evaluate interactome topological features, and typical examples are node degree and clustering coefficient, both based on the levels of connectivity of each node.

Both global and local connectivity can be explored by these methods, depending on the kind interactome analysis to be performed. The results may vary, as methods are based on different principles. For instance, the two main contributions to our work come from the application of two algorithms, CFinder ([7,8]) and MCODE [9]. Interestingly, they deal with network modularity through similar topological instruments, but achieve quite different outcomes; therefore, we based our analysis on them, while also evaluating other methods.

In parallel, a substantial heterogeneity of human interactome datasets has been generated depending on the underlying methods of identifying and characterizing protein interactions. Besides high-throughput approaches ([10-12]), in particular the curation of literature ([13-15]) and the provision of computational predictions ([16-18]) have allowed for the mapping of the human interactome.

Despite the impressive size of PPIN produced by the different approaches, their overall coverage remains limited. A general procedure to increase the coverage level is the integration of different interaction maps. However, recent analyses [19] have revealed that the class of integrated PPIN may display distinct functional characteristics and topological features. This evidence suggests that the analysis of integrated maps could be compromised by the heterogeneity which is fused into them, ending up with diverging modular maps.

To assess this possibility, we have defined integrated and also individual PPIN classes; as the latter have a source-specific characterization, they in turn generate disaggregated interactome datasets. In particular, datasets have been constructed from literature, orthology, and high-throughput experiments in an attempt to assess the variability of the modularity maps caused by the underlying source of interaction data. We have thus retrieved modules by various methods and from each interactome class, including the integrated one, and finally compared them by multiple validations.

A comparative evaluation of human protein interactome classes suggests that a scoring system should be available. We have proposed a confidence scoring method based on several sequential steps. Using gene expression data and gene annotation, we assigned initial confidence scores to PPIN modules. In order to calibrate the initial scores, information from GO http://www.geneontology.org/, MIPS (http://mips.helmholtz-muenchen.de/proj/ppi/, and KEGG http://www.genome.jp/kegg/) was subsequently utilized. Overall, the combined use of interactome classes, network clustering methods and additional multiomic sources allows for better characterization of the modularity map, and for an assessment of the influence of integration and disaggregation on the detected and validated modules.

The integrative approach requires specific tools for both analysis and validation, and is based on a qualitative and quantitative representation of a compilation of information from diverse biological sources. Specifically, we have focussed on how disaggregated and integrated interactome classes influence and characterize the detected modules. In addition, we addressed two related questions: Do clustering algorithms determine modularity maps? To what extent is the overlap or separation of modules induced by inherent data complexity or by capacity of the method to partition the data?

There are limitations in the current practice of interactome modularity detection and representation, and two main factors condition the analysis: the choice of the modularity-finding algorithm, and the choice of the interactome dataset with the related biological sources. In this article we provided evidence that quantifies the existing discrepancies between methods whose performance is comparatively evaluated over different interactome datasets. Simultaneous evaluation of both interactome methods and data may provide valuable guidance on the overall interpretability of modularity and likely lead to improved inference methods and models. The paper is organized in such way that module extraction algorithms are first introduced, then the structure of the available datasets is presented, and finally the results of a data integration approach are illustrated.

## Methods

### Detection of Modularity

Following many *Saccharomyces cerevisiae *(yeast) PPIN studies based on large-scale proteomic data (see for instance [20] and [21]), and concerned with modularity detection, similar analyses have been recently proposed for the human interactome. Not surprisingly, the latter presents more challenges and requires many efforts to substantially improve both coverage and accuracy ([10,22,23]).

Modularity is primarily studied to reveal the organization of a protein interactome into its constituent modules, to quantify their level of intra-cohesiveness and cross-communication, and to measure the overall partition quality based on biological grounds. To advance our understanding relatively to all these aspects, we performed extensive comparative analyses of newly compiled and carefully tracked interaction maps derived from the *Unified Human Interactome *(UniHI) database [24], which currently houses 253, 000 distinct interactions between over 22, 300 unique human proteins. Our study of the modular structures inhuman interactome involved two steps: one to compare the retrieved modules obtained from integrated and disaggregated human interactomes, and another to perform biological validation by multiple functional information sets and tissue-based gene expression data.

Notably, the inclusion of gene co-expression complies with earlier studies ([11,25,26]) in which the identification of protein modules was not based on interaction data only. We also believe that there is additional value in gene expression data to indicate the presence of the detected modules in certain tissues, as previous analysis of modular structures in the human protein interactome clearly demonstrated ([27,28]). Since gene co-expression and physically interacting proteins tend to be correlated for the human interactome, an integration might increase the reliablity of modules detected by computational algorithms. Thus, we performed a proteome-wide integration of expression and interaction data to assess the quality of the retrieved modules, which our results showed to be highly useful as an approach to the integration of complementary interaction maps.

In interaction maps, modules (also addressed as communities) represent densely connected sub-structures whose functions might be of biological relevance [29]. They recur frequently and with variable size in protein interactomes. Several detection methods [30] have been proposed to identify modules based on different principles, but with the common shortcoming of achieving only a limited resolution spectrum (i.e all the possible module sizes) when applied to large networks ([31,32]).

Interestingly, while the modules are expected to represent highly related functions, it has been observed [33] that known pathways in metabolic networks do not correspond to top-scoring modules, as large pathways are composed of smaller units which are mixtures of sub-structures associated to different pathways. The same considerations may hold for more general protein pathways where hub-like proteins ([34,35]) are usually essential ([36-38]) in the network and maintain sparse links between different modules, while other protein regulators characterize modules of smaller size. Since protein interactomes usually show heterogeneity in their module distribution with regard to size, we elucidated this aspect in the next section, in relation to both methods and interactome data.

### Proposed Algorithms

A novel aspect of our study is the comparative analysis of interactome aggregation and disaggregation effects. For the comparison, we first assembled three different human PPIN solely derived by manually-curated interactions from the literature (Lit-PI, 9321 proteins and 37690 interactions), by computationally predicted interactions using orthology (Ortho-PI, 5091 proteins and 13639 interactions), and by high-throughput protein interactions (HTP-PI, 2957 proteins and 5899 interactions). Finally, we fused them to an integrated dataset referred as Int-PI (11267 proteins and 54613 interactions).

In order to extract the modules (see Table 1 for the complete summary), we applied two different network clustering algorithms, C finder and MCODE, to all the disaggregated and the integrated datasets, and then compared the retrieved modules. These two deterministic methods are topology-based and centered on 'cliques' as the reference entities to identify modules. Therefore, they both depend on local node-connectivity due to the definition of clique, i.e. a maximally connected graph or an induced sub-graph which is a complete graph, or equivalently a graph with every pair of distinct nodes connected by a link. In contrast, both algorithms differ considerably with regard to their computational implementation strategies (details are reported in the Appendix). We considered the deterministic definitions of cohesive subgraphs from both MCODE and CFinder as possible ways to explore the degree of separation versus overlapping of protein groups through their complex underlying clique structure.

**Table 1:** Modules by method and dataset

	**CF comm**.	CF-range of *k*	2-core MC	MC-range of *k*	**MaxMod comm**.	**Walktrap comm**.
Int	1096	3 - 17	136	2 - 19	179	314
Lit	779	3 - 11	101	2 - 10	170	254
Ortho	397	3 - 17	141	2 - 18	211	250
HTP	61	3 - 4	11	2 and 3	129	151

Summary of CFinder, MCODE, MaxMod and Walktrap modules for UniHi datasets.

We have then applied a third method [39] to our datasets. *Q*-modularity, whose maximization is here addressed as the MaxMod approach, aims at partitioning a network in modules that emphasize deviations from purely random dynamics. In particular, for a partition in *N *modules, with *e*_*ij *_establishing links between modules *m*_*i *_and *m*_*j *_, and given *E*_*i *_= ∑_*j*_*e*_*ij *_and *Tr*[*E*] = ∑_*i*_*e*_*ij *_, the modularity function can be formulated (see also [40-42]) as follows:(1)

Thus, *Q *associates proportions of links within each module with proportions of all links (i.e. expected in the whole network, within and between modules); equivalently, it compares observed modular to inherently non-modular network architectures. Intuitively, a good modular partition would lead to high values for the trace, and *Q *would approach 1; vice versa, a large presence of random links between nodes (i.e. poor modularity) would make *Q *approaching 0. In general, a modular partition shows dense intra-modular links and sparse inter-modular links. Equivalently, the detected modularity structure presents a few local maxima capturing the most relevant information of the internal network organization.

**Figure 1:**
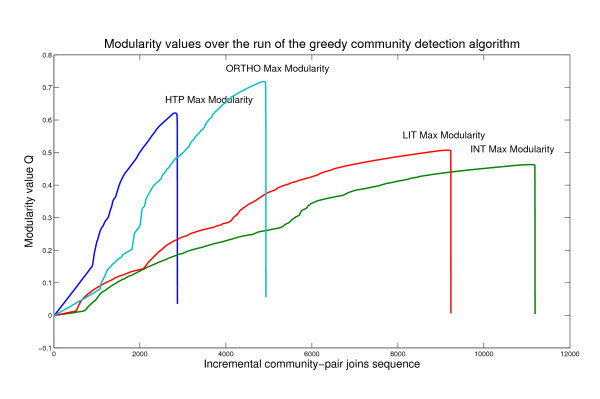
**MaxMod Patterns**. Comparative MaxMod performance for the set of interactions networks studied: in agreement with known studies, values approximately ranging in 0.3 - 0.7 suggest modular structure. Software freely available through R -*igraph *package, *fastgreedy.community *function- then plotted from Matlab.

MaxMod leverages on a greedy optimization algorithm that starts by modules of one element, i.e. each node, and iterates a merging process designed to join the module pair whose amalgamation creates the largest modularity increase (see Figure 1). As said, the optimization function is defined to be zero in one case, when the fraction of within-module links is equal to what we would expect for a randomized network of equivalent size. Otherwise, non-zero values indicate deviation from randomness (a value around 0.3 is commonly retained a lower bound for the presence of modular structure).

We note that the induced sparsity implies a poor identification power with regard to the resolution spectrum, especially for small and intermediate module sizes. Due to the retrieval of coarse resolution modules, whose large sizes depend on incremental merging of small modules, a weakness of the MaxMod approach concerns its possible interpretation in biological applications. In addition, more reasons of concern exist with reference to methodological aspects. First, more than one partition could reach the maximal modularity (local maxima). Second, the modularity definition could reveal only some groups (due to bias). Third, as modularity calculation is sensitive to noise, an optimal partition may not be achieved. Consequently, the MaxMod sub-optimality effect of limiting the coverage for the network resolution spectrum requires investigation when all module sizes may in principle count.

Then, we extended the analysis to a random walk (RW) search, and thus considered a stochastic optimization approach centered on the definition of a distance that measures structural similarity between nodes. We have thus applied the *Walktrap *algorithm [43], freely available in the *igraph *R package, in order to know whether a resolution refinement is possible in the modularity structure by allowing more chances of escaping from high-density regions. RW introduces probabilistic elements in the hierarchical scheme underlying most of the algorithms, and can be compared with the previous algorithms.

Since our two benchmark methods rank their outputs according to an internal scoring system, we selected only highly ranked sub-networks for further investigation (more details appear in the Appendix, Method parameterization section). Specifically, we refer to 65 groups from CFinder's results (21 for Int-PI, 14 for Lit-PI, 21 for Ortho-PI and 9 for HTP-PI), and to 45 groups from MCODE's results (14 for Int-PI, 13 for Lit-PI, 14 for Ortho-PI and 4 for HTP-PI).

We also considered network inference aspects involving protection against random effects, such as design of randomization schemes and generation of possible null models. The matter is still controversial [44], and in our study we present distinct interactome data and methods that prevent from conceiving a unified scheme, but some remarks are as follows.

MCODE was designed and tested to be robust; for instance, it was shown in [9] that large-scale and noisy experimentally derived interactions do not remarkably affect the prediction of complexes by MCODE, while [3] referred to increased robustness compared to other methods in terms of negative control and unassigned nodes.

As for CFinder, the randomization effects have been evaluated on a large set of generated random networks [19], in particular on homogeneous scale-free networks. The results indicated that the observed number of k-cliques (with *k *> 5) is indeed highly significant, while for small-sized communities there is an increased chance of results that become close to random outcomes.

Consequently, the retrieved number of CFinder communities may be indeed overestimated, likewise the real distance between the extremes (MCODE and CFinder) of the resolution scale emphasized by the distribution of module sizes. In turn, such evidence justifies our approach based on thresholding. Moreover, recent results [45] suggest that both algorithms and datasets should be considered in order to establish acceptable solutions such as hierarchies of null models.

### Confidence Scoring

Following an original approach [46] previously proposed for an integrated interactome analyzed by CFinder, we have combined the module ID extracted from each disaggregated interactome with the variables listed and described in Table 2, and with the compendium of gene co-expression measurements generated from the Human Gene Atlas data source http://biogps.gnf.org. Since we do not focus in our study on a particular tissue, we have chosen to utilize a compendium of gene expression profiles comprising a large number of tissues and generated on a single platform to minimize experimental variability.

**Table 2:** Variable selection method

variable	description
*n*	number of proteins included in module
*n.ex*	number of proteins in module included in the Human Gene Expression Atlas dataset
*ex.mean*	average expression scores in human tissues, measured for the Gene Atlas
*cor.mean*	module pairwise Spearman correlation of tissue expression
*mf.mean*	mean "co-annotation" for GO molecular function
*bp.mean*	mean "co-annotation" for GO biological process
*cc.mean*	mean "co-annotation" for GO cellular component

With regard to the Human Gene Expression Atlas dataset, the available link is at http://biogps.gnf.org

The term "co-annotation" refers to the similarity of GO annotation of interacting proteins, based on a comparison of the associated GO graphs. The size of the intersection of the graphs divided by the size of their union was used as a similarity measure. The implementation of the variable selection method is elucidated in Table 3, where GO annotation results are presented for some modules selected by MCODE and CFinder. In particular, we have combined the variable selection approach with a scoring procedure based on thresholding in order to assign confidence measures to the modules (an overview of the applied scoring system is provided apart).

**Table 3:** GO-based annotation results of CFinder and MCODE modules

	module ID	n	n.ex	ex.mean	cor.mean	mf.mean	bp.mean	cc.mean
High	Int-2	5	5	8.33	0.22	**0.76**	**0.7**	**0.7**
	Int-3	5	1	8.54	NA	**0.85**	**0.88**	**0.84**
	Int-4	9	7	9.56	0.43	**0.7**	**0.81**	**0.92**
	Int-7	17	13	8.88	0.17	**0.63**	**0.58**	**0.66**
	Lit-2	5	5	8.33	0.22	**0.76**	**0.7**	**0.7**
	Lit-5	9	9	9.62	0.42	**0.59**	**0.68**	**0.66**
	Lit-9	8	8	9.02	0.62	**0.85**	**0.92**	**0.82**
	Lit-11	10	9	8.17	0.3	**0.58**	**0.74**	**0.61**
	Ortho-8	15	11	8.9	0.15	**0.68**	**0.57**	**0.67**
	Ortho-12	10	9	10.73	0.59	**0.83**	**1**	**0.98**

Medium	Int-18	18	16	9.55	***0.54***	0.45	**0.51**	**0.7**
	Ortho-18	17	15	9.65	***0.58***	0.49	**0.6**	**0.72**

Low	Int-13	13	11	8.79	0.39	0.45	**0.73**	**0.96**
	Lit-4	16	11	7.95	0.24	0.28	**0.52**	**0.58**
	Lit-14	11	10	8.79	0.39	0.4	**0.72**	**0.95**
	Ortho-7	8	7	9.48	0.41	0.48	**0.56**	**0.61**

**MCODE**	module ID	n	n.ex	ex.mean	cor.mean	mf.mean	bp.mean	cc.mean

High	Int-5	9	7	9.56	0.43	**0.7**	**0.81**	**0.92**
	Lit-4	7	7	8.99	0.13	**0.89**	**0.79**	**0.71**
	Ortho-4	12	9	9.37	0.31	**0.51**	**0.59**	**0.54**
	Ortho-5	9	7	12.92	0.77	**0.65**	**0.78**	**0.71**

Low	Lit-11	9	8	8.34	0.24	0.4	**0.59**	**0.69**
	Lit-13	22	20	8.81	0.32	0.33	**0.5**	**0.63**
	Ortho-2	6	5	8.08	0.29	0.41	**0.78**	**0.73**
	Ortho-10	11	9	8.38	0.38	0.39	**0.52**	**0.67**

GO-based annotation results of CFinder andMCODE modules: High (bold font for every GO category), Medium (*cor.mean *≧ 0.5), and Low (*cor.mean *< 0.5) confidence groups.

Based on the last three average quantities listed as *mf.mean, bp.mean, cc.mean*, we have made an even finer selection by retaining only the modules with *mf.mean*, *bp.mean *and *cc.mean *values ≧ 0.5, our thresh old (indicated in bold font in Table 3). We call them *High Confidence *modules. According to [47], two interacting proteins must be close to each other in a permanent or transient manner; proteins in the same complex should be localized at the same cellular compartment, while the non-interacting proteins should likely be just transient, if not spuriously present (see also [48]). Thus, co-localization explains our rationale for keeping *cc.mean *≧ 0.5. Similarly, we apply the same numeric threshold for the other two GO categories, in order to characterize the functional association strength of each module.

Correspondingly, we have defined *Medium Confidence *and *Low Confidence *modules according to two criteria. First, at least two over three GO thresholds (*bp.mean *and *cc.mean*) had to be satisfied. Second, modules satisfying an expression correlation threshold, i.e. *cor.mean *≧ 0.5, retained a medium confidence level, while modules with *cor.mean *< 0.5 were assigned a low confidence level (further evidence for all modules excluded by this selection is reported as supporting information.

In summary, due to the strong indications that co-localization correlates at the transcriptional level with co-expression, and in turn with biological processes and molecular functions, protein module selection has been performed through the entire set of GO categories. Indeed, interacting protein pairs in a complex tend to show mRNA co-expression [25] reinforcing protein modularity maps [26]. Further evidence is also available from experimentally derived data [11] and tissue-specific interactome analysis [28].

We then evaluated multiple validation sources in both qualitative and quantitative terms, and also performed' scoring calibration' (evidence is also available in Additional file 1, Additional file 2, Additional file 3 and Additional file 4). Calibration consists of three main steps aimed at refining the described scoring system, and the corresponding confidence levels assigned to modules. As a first step, we proceeded through GO annotation by ranking modules according to computed FDR-corrected p-values (full details are in the previously addressed online files) in order to assess the pre-assigned confidence levels (as from Table 3).

As a second step, we considered annotated complexes to measure the overlap scores (of three types, see Additional file 3). We mapped every selected module over both MIPS and Reactome domains to exploit more coverage (CORUM - http://mips.helmholtz-muenchen.de/genre/proj/corum[49] and also COFECO - http://piech.kaist.ac.kr/cofeco[50] were used).

Finally, we looked at pathway enrichment through p-values (see Additional file 4) and performed a qualitative evaluation aimed to characterize each module according to inherently cohesive (i.e. intra-modular, self-contained) versus cross-talk (i.e. inter-modular, communicative) dynamics. A very interesting aspect to investigate is indeed a characterization for more than just sparsely interacting cohesive modules, as the latter may be heavily involved in cross-talks according to 'connector groups' [51], i.e. involved in the same connecting functional role by showing a variable degree of cohesiveness.

A final remark is with regard to the assignment of our confidence scores and concerns the influence of thresholding. In particular, our results are in part sensitive to the selected fixed thresholds (among the ones which were considered). Repeated testing (data not shown) has suggested the best possible threshold choice from the available data. Another consideration is about the whole sequence of steps required to implement our multilayer approach, which reflects the scoring system in terms of validation sources. We have added pathway to other GO-based inference and MIPS analysis in order to validate our modules, and emphasize their permanent versus transient characterization. Last, by casting this framework in a protein pathway context, we could compare the detected modules with pre-assigned confidence scores against other multiple validation approaches (see the points discussed below about protein pathways).

### Algorithm Performance Measures

Precision and recall [52] are two well-known measures to test the performance of algorithms. In Eq. (2) context, precision refers to the predicted interacting pairs that match true positives, recall refers to interacting pairs identified by the algorithm out of all the possible known ones. We report both precision and recall formulas (computed for both MCODE and CFinder within each module), given *tp *as the true positives, *fp *as the false positives, and *fn *as the false negatives, as follows:(2)

Then, we report in Eq. (3) precision and recall computed again for both the methods, but this time across the modules in each dataset (we mark with a 'prime' both quantities). Thus, MM and PM represent the significantly matched and the predicted modules, respectively, while MC and KC represent the well matched and the known MIPS complexes, respectively. Following [53], it is important to note that MM (a module-based measure) is not necessarily equal to MC (a complex-based measure), because the same complex can be reflected by multiple detected modules. We have reported in detail evidence for the numerous well-studied complexes detected by clustering methods.(3)

## Results and Discussion

Given the list of methodological steps previously described, we first report numerical outcomes and then proceed with the assessment of our results based on each performed validation.

### Numerical Evidence

The retrieved modules indicate that CFinder has identified 2333 modules (named as "communities"), which are mostly based on 3- and 4- cliques (*k *= 3 - 355; *k *= 4 - 200). MCODE has instead generated 389 modules (named as "cores", where a "2-core" is for a clique size of 2, thus the least stringent choice in terms of connectivity which is accounted for). Results for varying ranges of *k *have also been reported for both methods, and the algorithmic details and parameters explained in Materials and Methods of the Appendix.

Although not exhaustive, this comparative evaluation is useful to define the inherent resolution power of each method, and elucidate the specific modularity associated to each interactome class. The modules result dependent on both methods and data, and can be compared in terms of resolution and distributional aspects. In particular, MCODE and CFinder represent quite far extremes in the resolution range allowed by the examined data, while both greedy and stochastic learning approaches stand in between these extremes and thus depict only intermediate modularity maps.

**Figure 2:**
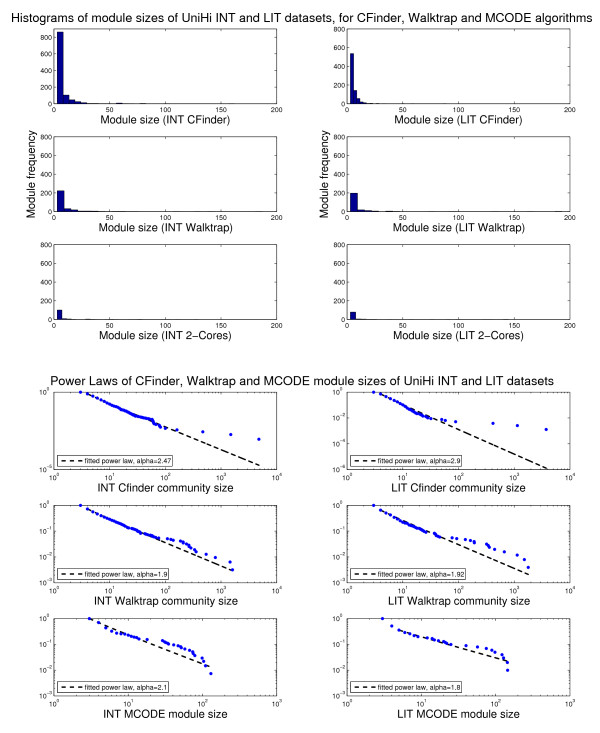
**Histograms of module sizes (Int-PI and Lit-PI) and Power Laws**. Distributional aspects of module sizes for Int-PI and Lit-PI datasets, and corresponding power laws (from Matlab scripts *plfit.m, plplot.m *in http://www.santafe.edu/~aaronc/powerlaws). Values for *alpha *approximately between 2 and 3 are typically expected.

Consequently, we have corroborated the analysis by investigating the resolution at which the modules are detected by each method and each dataset (evidence from two datasets has been reported in Figure 2, and from other datasets as supporting information). With regard to the number of retrieved modules in each interactome class, MaxMod converges to MCODE (except for HTP), probably due to the underlying hierarchical structure present in both schemes. The inherent modularity can remain partially latent when the MaxMod method is applied to protein interactions. Thus, a small number of final modules can be found, but mainly due to the module merging effects rather than biological structure. As a result, more sparsity implies lack of detection power for small and intermediate module sizes, whose overlaps are instead observed with CFinder.

We have then provided evidence for the distribution of module sizes by the histograms obtained from the frequency with which each module size appears. As a result of its overlapping structure, CFinder covers the interactome resolution spectrum of module sizes to the highest degree, thus exploring more extensively the inherent module heterogeneity. Through a RW-based search, a distributional change can be observed, but we still found that the resolution spectrum remained comprised between those of MCODE and CFinder. Furthermore, in Figure 2 we have combined the resolution evidence for both the Int-PI and the Lit-PI datasets with the corresponding power laws, given that from *x *drawn from a certain probability distribution *p*(*x*), a power law is observed when *p*(*x*) ∝ *x*^-α ^with α the scaling parameter [54].

### Multilayer Validation

#### Pathway Analysis

An important outcome of our multilayer approach is the possibility of analyzing each protein module based on a grid of confidence levels. One step is designed to use the *KEGG *Pathway Database [55], which has been interrogated by queries of proteins from each extracted module and then annotated to a specific pathway (Table 4).

**Table 4:** Pathways

CFinder	module ID	Protein IDs	Pathways
High	Int-2	-	na
	Int-3	-	na
	Int-4	-	na
	Int-7	POLR3G POLR3A POLR3 H POLR1A ZNRD1 POLR1D POLR2C POLR2E POLR2 H POLR2L POLR1B POLR3GL POLR1C	hsa03020 RNA polymerase
	Lit-2	-	na
	Lit-5	POLR2A POLR2C POLR2 D POLR2E POLR2G POLR2 H POLR2L	hsa03020 RNA polymerase
	Lit-9	LSM1 LSM2 LSM3 LSM4 LSM5 LSM6 LSM7 LSM1 LSM2 LSM3 LSM4 LSM5 LSM6 LSM7	hsa03018 RNA degradation hsa03040 Spliceosome
	Lit-11	SKIV2L2 EXOSC2 EXOSC4 EXOSC5 EXOSC6 EXOSC7 EXOSC8 EXOSC9	hsa03018 RNA degradation
	Ortho-8	POLR3G POLR3A POLR3 H POLR1A ZNRD1 POLR1 D POLR2E POLR2F POLR2 H POLR2L POLR1B POLR3GL POLR1C	hsa03020 RNA polymerase
	Ortho-12	PSMA1 PSMA2 PSMA4 PSMA6 PSMA7 PSMB1 PSMB2 PSMB3 PSMB5	hsa03050 Proteasome

Medium	Int-18	PSMA1 PSMB2 PSMC2 PSMC3 PSMD11 PSMD12 PSMD13 PSMD6	hsa03050 Proteasome
	Ortho-18	PSMA1 PSMB2 PSMC2 PSMC3 PSMD11 PSMD12 PSMD13 PSMD6	hsa03050 Proteasome

Low	Int-13	TAF1 TAF2 TAF4 TAF5 TAF6 TAF7 TAF9 TAF10 TAF12 TAF13 TBP	hsa03022 Basal transcription factors
	Lit-4	MYC RUVBL1	hsa04310 Wnt signaling pathway
	Lit-14	TAF1 TAF2 TAF4 TAF5 TAF6 TAF7 TAF9 TAF10 TAF12	hsa03022 Basal transcription factors
	Ortho-7	EXOSC2 EXOSC3 EXOSC4 EXOSC5	hsa03018 RNA degradation

**MCODE**	module ID	Protein IDs	Pathways

High	Int-5	-	na
	Lit-4	-	na
	Ortho-4	DDX6 LSM1 LSM2	hsa03018 RNA degradation
	Ortho-5	RPL3 RPL12 RPL27A RPS2	hsa03010 Ribosome

Low	Lit-11	-.	na
	Lit-13	TAF1 TAF2 TAF4 TAF5 TAF6 TAF7 TAF9 TAF10 TAF12 HDAC1 HDAC2	hsa03022 Basal transcription factors hsa04110 Cell cycle
	Ortho-2	TAF9B TAF5 TAF6 TAF12	hsa03022 Basal transcription factors
	Ortho-10	PPP1CA PPP1CB	hsa04720 Long-term potentiation hsa03018 RNA degradation hsa04810 Regulation of actin cytoskeleton hsa04910 Insulin signaling pathway hsa04510 Focal adhesion

Pathways results for CFinder and MCODE High, Medium and Low confidence modules (KEGG source [55], *na*: not annotated). See also: Figure 9-12.

With CFinder, High Confidence modules have identified in most cases the same *hsa03020 *pathway, thus behaving similarly across the different datasets. The same occurs for the *hsa03022 *pathway in both MCODE's and CFinder's Low Confidence modules.

Overall, a quite strong pathway characterization is found for the different confidence levels, with some differences: the effect is more method-dependent for the High Confidence cases, thus the variation occurs across the datasets, and to a larger extent compared to the Low Confidence case.

Disaggregated datasets lead to an evaluation of both intra-dependence and inter-dependence relationships which can arise within and between the datasets, respectively. Intra-dependence addresses specific structural features and biases characterizing each dataset, i.e. types of correlation of an endogenous nature. Inter-dependence addresses overlaps or redundancies among different datasets, which are of relevance in addition to their distinct information contents.

Similarly, even if in relation to another scale, binary interactions have been considered either intra-complex, i.e. occurring within protein complexes, or non intra-complex, because not found to belong to one of them in particular. The latter category naturally links to transient interactions, while permanent interactions are typically intra-complex dynamics. We have presented examples of permanent and transient modules, after analyzing protein complexes and how the retrieved modules could map to them.

#### Functional Annotation by Gene Ontology

**Figure 3:**
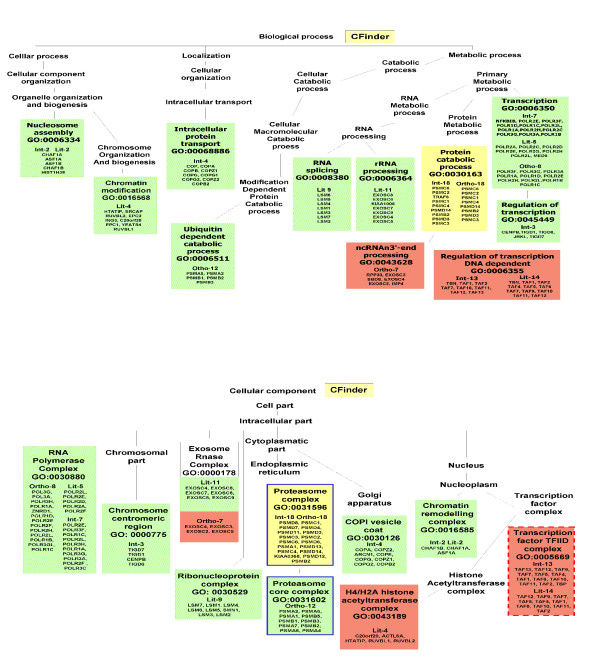
**GO-annotation results - Cfinder**. Representation of GO-annotation results concerning biological process and cellular component of CFinder modules, along the GO trees. In green, yellow and red colors are High, Medium and Low Confidence modules, respectively. Blue/red frame indicates permanent/transient complexes.

**Figure 4:**
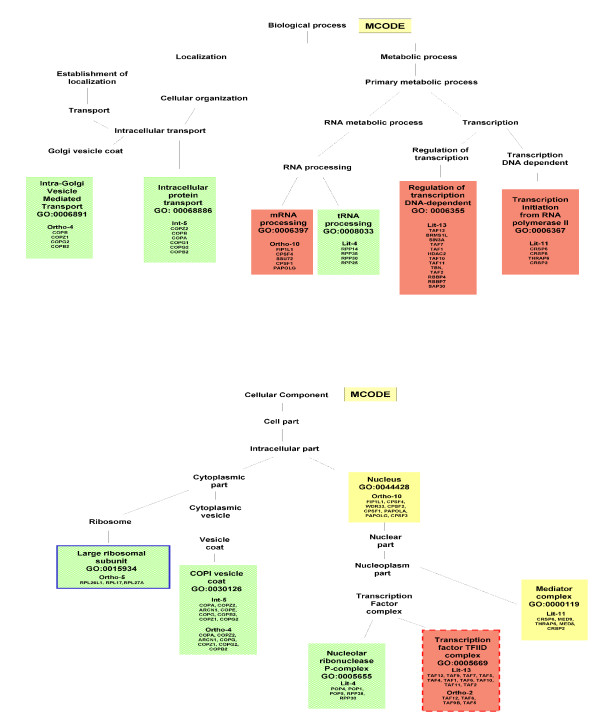
**GO-annotation results - MCODE**. Representation of GO-annotation results concerning biological process and cellular component of MCODE modules, along the GO trees. In green, yellow and red colors are High, Medium and Low Confidence modules, respectively. Blue/red frame indicates permanent/transient complexes.

We have carried out GO annotation in order to identify complexes that account for both biological process (Table 5) and cellular component (Table 6), and reported the complete annotation for both methods in Figure 3 and Figure 4.

**Table 5:** GO-based Biological Process annotation

CFinder	module ID				
High	Int-2	CHAF1A ASF1A ASF1B CHAF1B HIST1H3E	nucleosome assembly ⋆	5/5	1.23e-10
	Int-3	CENPB TIGD1 TIGD6 JRKL TIGD7	regulation of transcription	5/5	0.00040
	Int-4	COPE COPA COPB COPZ1 COPG COPG1 COPG2 COPZ2 COPB2	intracellular protein transport	9/9	6.97e-12
	Int-7	NFKBIB POLR2E POLR3F POLR1D POLR1C POLR2L POLR1A POLR2H POLR2C POLR3G POLR3A POLR1B	transcription ◆	12/17	6.01e-08
	Lit-2	CHAF1A ASF1A ASF1B CHAF1B HIST1H3E	nucleosome assembly ⋆	5/5	1.23e-10
	Lit-5	POLR2A POLR2C POLR2D POLR2E POLR2G POLR2H POLR2L MED9	transcription ◆	8/9	8.88e-07
	Lit-9	LSM6 LSM5 LSM4 LSM1 LSM3 LSM7 LSM2 SMN1	RNA splicing	8/8	7.48e-17
	Lit-11	EXOSC8 EXOSC6 KIAA1008 EXOSC7 EXOSC9 EXOSC4 EXOSC5	rRNA processing	7/10	4.73e-17
	Ortho-8	POLR3F POLR3G POLR3A POLR1A POLR1D POLR2E POLR2H POLR2L POLR1B POLR1C	transcription ◆	10/15	2.95e-06
	Ortho-12	PSMA8 PSMA2 PSMB1 PSMB2 PSMB3	ubiquitin-dependent ● protein catabolic process	5/10	9.49e-07

Medium	Int-18	PSMC6 PSMC2 TRAF6 PSMC1 PSMC4 PSMD14 PSMB2 PSMD3 PSMC3	protein catabolic process ●	9/18	1.12e-10
	Ortho-18	PSMC6 PSMC2 PSMC1 PSMC4 PSMD14 PSMB2 PSMD3 PSMC3	protein catabolic process ●	8/17	2.87e-09

Low	Int-13	TBNTAF1 TAF2TAF7 TAF10 TAF11 TAF12 TAF13	**regulation of transcription, DNA-dependent**	8/13	3.35e-07
	Lit-4	HTATIP SRCAP RUVBL2 EPC2 ING3 C20orf20 EPC1 YEATS4 RUVBL1	chromatin modification	9/16	6.61e-16
	Lit-14	TBN TAF1 TAF2 TAF4 TAF5 TAF6 TAF7 TAF9 TAF10 TAF11 TAF12	**regulation of transcription, DNA-dependent**	7/11	3.34e-06
	Ortho-7	RPP30 EXOSC3 SBDS EXOSC4 EXOSC5 IMP4	ncRNA processing	6/8	2.53e-12

					

**MCODE**	module ID	Protein IDs	Biological Process	cluster freq.	p-value

High	Int-5	COPZ2 COPB COPA COPG1 COPG2 COPB2	intracellular protein transport	6/9	6.97e-12
	Lit-4	RPP14 RPP38 RPP30 RPP25	tRNA processing	4/7	5.17e-10
	Ortho-4	COPB COPZ1 COPG2 COPB2	intra-Golgi vesicle-mediated transport	4/12	5.24e-10
	Ortho-5		No significant terms		

Low	Lit-11	CRSP6 CRSP8 THRAP6 CRSP2	transcription initiation from RNA polymerase II promoter	4/9	1.92e-08
	Lit-13	TAF12 BRMS1L SIN3A TAF7 TAF1 HDAC2 TAF10 TAF11 TBN TAF2 RBBP4 RBBP7 SAP30	**regulation of transcription, DNA-dependent**	13/22	7.12e-12
	Ortho-2	-	NA	-	-
	Ortho-10	FIP1L1 CPSF4 SSU72 CPSF1 PAPOLG	mRNA processing	5/11	5.71e-09

GO-based Biological Process annotation results for CFinder and MCODE High, Medium and Low confidence modules. Marked with a symbol (⋆, ▲, ●) are modules with the same annotation; identical results across the two methods are in bold font. P-values are described in Additional files 2 and 10. Cluster frequency indicates, for a given module, the number of genes annotated over the number of all included genes.

**Table 6:** GO-based Cellular Component annotation

CFinder	module ID	Protein IDs	Biological Process	**cluster freq**.	p-value
High	Int-2	CHAF1B CHAF1A ASF1A	chromatin remodeling complex ⋆	3/5	0.000001
	Int-3	TIGD7 TIGD1 CENPB TIGD6	chromosome centromeric region	4/5	0
	Int-4	COPA COPZ2 ARCN1 COPE COPG COPZ1 COPG2 COPB2	**COPI vesicle coat**	8/9	1.98E-025
	Int-7	POLR2E POLR3F POLR1C POLR2L POLR3 H POLR1A POLR3G POLR3A POLR2F POLR3C	RNA polymerase complex ◆	10/17	6.95E-025
	Lit-2	CHAF1B CHAF1A ASF1A	chromatin remodeling complex ⋆	3/5	0.000001
	Lit-5	POLR2L POLR2E POLR2 D POLR2A POLR2F	RNA polymerase complex ◆	5/9	0
	Lit-9	LSM7 LSM1 LSM4 LSM6 LSM5 SMN1 LSM3 LSM2	ribonucleoprotein complex	8/8	0
	Lit-11	EXOSC4 EXOSC2 EXOSC8 EXOSC7 EXOSC6 EXOSC5 EXOSC9	exosome (Rnase complex)	7/10	1.61E-022
	Ortho-8	POLR2E POLR3F POLR1C POLR2L POLR3 H POLR1A POLR3G POLR3A POLR2F POLR3C POLR1A POLR3G POLR3A POLR2F POLR3C	RNA polymerase complex ◆	10/15	1.42E-025
	Ortho-12	PSMA2 PSMA6 PSMA1 PSMB5 PSMB1 PSMB3 PSMA7 PSMB2 PSMA8 PSMA4	proteasome core complex ●	10/10	2.89E-027

Medium	Int-18	PSMD8 PSMC1 PSMD7 PSMD4 PSMD11 PSMD3 PSMC3 PSMC2 PSMC6 PSMD6 PSMA1 PSMD13 PSMC4 PSMD14 KIAA0368 PSMD12 PSMB2	proteasome complex ●	17/18	1.06e-41
	Ortho-18	PSMD8 PSMC1 PSMD7 PSMD4 PSMD11 PSMD3 PSMC3 PSMC2 PSMC6 PSMD6 PSMA1 PSMD13 PSMC4 PSMD14 KIAA0368 PSMD12 PSMB2	proteasome complex ●	17/17	1.60e-42

Low	Int-13	TAF13 TAF12 TAF9 TAF7 TAF5 TAF4 TAF1 TAF6 TAF10 TAF11 TAF2 TBP	**transcription factor TFIID complex**	12/13	5.58e-36
	Lit-4	C20orf20 ACTL6A HTATIP RUVBL1 RUVBL2	H4/H2A histone acetyltransferase complex	5/16	5.80e-15
	Lit-14	TAF12 TAF9 TAF7 TAF5 TAF4 TAF1 TAF6 TAF10 TAF11 TAF2	**transcription factor TFIID complex**	10/11	1.38e-29
	Ortho-7	EXOSC4 EXOSC3 EXOSC2 EXOSC5	exosome (Rnase complex)	4/8	1.31e-11

					

**MCODE**	module ID	Protein IDs	Biological Process	cluster freq.	p-value

High	Int-5	COPA COPZ2 ARCN1 COPE COPG COPB2 COPZ1 COPG2	**COPI vesicle coat**	8/9	1.98E-025
	Lit-4	POP4 POP1 POP5 RPP38 RPP30	nucleolar ribonuclease P-complex	5/7	6.91E-017
	Ortho-4	COPA COPZ2 ARCN1 COPG COPZ1 COPG2 COPB2	**COPI vesicle coat**	7/12	3.49E-020
	Ortho-5	RPL3 RPLP0 RPL26L1 RPL12 RPL17 RPL10 RPS2 RPL27A	large ribosomal subunit	8/9	1.78e-11

Low	Lit-11	CRSP6 MED9 THRAP6 MED8 CRSP2	S mediator complex	5/9	1.18e-13
	Lit-13	TAF12 TAF9 TAF7 TAF5 TAF4 TAF1 TAF6 TAF10 TAF11 TAF2	**transcription factor** **TFIID complex**	10/22	4.81e-25
	Ortho-2	TAF12 TAF6 TAF9B TAF5	**transcription factor** **TFIID complex**	4/6	4.01e-10
	Ortho-10	FIP1L1 CPSF4 WDR33 CPSF2 CPSF1 PAPOLA PAPOLG CPSF3	nucleus	8/11	0.00206

GO-based Cellular Component annotation results for CFinder and MCODE High, Medium and Low confidence modules. Marked with a symbol (⋆, ▲, ●) are modules with the same annotation; identical results across the two methods are in bold font. P-values are described in Additional files 2 and 10. Cluster frequency indicates, for a given module, the number of genes annotated over the number of all included genes.

Our annotation emphasizes the inherent modularity in the available datasets, as shown by mapping the extracted modules to known complexes. The Int-PI dataset overlaps sometimes with the Lit-PI and the Ortho-PI datasets, but also extracts modules which are uniquely detected (and not appearing in disaggregated datasets).

Some examples are: Int-2, which overlaps with Lit-2 (*Chromatin remodelling complex*); Int-7, which overlaps with Ortho-8 (*RNA polymerase complex*); Int-18, which overlaps with Ortho-18 (*Proteasome complex*). In other cases the Int-PI dataset extracts non-overlapping modules, such as Int-3 (*Chromosome centromeric region*), or Int-4 (*COPI vesicle coat*).

By comparison of the disaggregated datasets with the Int-PI dataset, we have identified some non-overlapping modules, such as Lit-4 (*H4/HA2 histone acetyltranferase complex*) or Lit-11 (*Exosome*). This is evident especially in the High Confidence modules.

With the MCODE method we have selected only one Int-PI module, Int-5 which overlaps with Ortho-4. In disaggregated datasets, overlapping Lit-13 and Ortho-2 (*Transcription factor TFIID complex*) modules are extracted.

Overall, the extraction of overlapping modules Int-13 and Lit-14 with CFinder, and Lit-13 and Ortho-2 with MCODE) involved the *Transcription factor TFIID complex*, while other cases (Int-4 with Cfinder, and Int-5 and Ortho-4 with MCODE) involved *COPI vesicle coat*. However, separated complexes were retrieved as well.

#### MIPS Mapping

After KEGG and GO annotations, we have validated our modules (Table 7) by mapping them to known complexes in MIPS [49]. Such step delivers comparative algorithmic performance across different interactome classes.

**Table 7:** Precision and Recall

*CFinder* modules	Confidence level	MIPS complex name	p-value	Precision	Recall
Lit-2	High	Chromatin assembly complex (CAF-1 complex)	0.000001	0.4	0.4
Lit-5	High	RNA polymerase II core complex	0	0.89	0.67
Lit-9	High	LSM 1-7 complex	0	0.88	1
Lit-11	High	Exosome	1.61e-022	0.7	0.7
Lit-14	Low	TFIID subcomplex	1.38e-29	1	1
Lit-4	Low	TIP60 HAT complex	5.80e-15	0.25	0.8
Ortho-8	High	RNA polymerase II core complex	1.42e-025	0.24	0.34
Ortho-12	High	20S proteasome	2.89e-027	0.73	0.57
Ortho-18	Medium	PA700 complex	1.60e-42	0.72	0.65
Ortho-7	Low	Exosome	1.31e-11	0.5	0.4
Int-2	High	Chromatin assembly complex	o.000001	0.4	0.4
Int-3	High	CEN complex	0	0.2	0.03
Int-4	High	-	-	-	-
Int-7	High	RNA polymerase II core complex	6.95e-025	0.26	0.42
Int-18	Medium	PA 700 complex	1.06e-41	0.6	0.6
Int-13	Low	TFIID subcomplex	5.58e-36	0.38	1

**Literature**				0.83	0.86
**Orthology**				0.25	0.25
**Integrated**				0.4	0.69

*MCODE* modules	**Confidence level**	**MIPS** complex name	**p-value**	**Precision**	**Recall**

Lit-4	High	Rnase/Mrp complex	6.91e-017	0.86	0.6
Lit-11	Low	MED18-MED20-MED29 mediator subcomplex	1.18e-13	0.11	0.34
Lit-13	Low	TFIID subcomplex	4.81e-25	0.24	1
Ortho-4	High	-	-	-	-
Ortho-5	High	60S ribosomal subunit, cytoplasmic	1.78e-11	0.78	0.15
Ortho-2	Low	TFIID subcomplex	4.01e-10	0.43	0.6
Ortho-10	Low	Cleavage and polyadenylation factor (CPSF)	0.00206	0.46	1
Int-5	High	-	-	-	-
Int-3	-	Nop56p-associated pre-rRNA complex	4.88e-17	0.38	0.05
Int-10	-	Arp2/3 complex	1.18e-19	0.6	0.86
Int-14	-	39S ribosomal subunit, mitochondrial	8.92e-17	0.23	0.88

**Literature**				0.67	0.25
**Orthology**				0.5	0.23
**Integrated**				0.34	0.13

Precision and Recall values for each module selected from each dataset with various confidence levels. Modules without indicated confidence level have been successively recovered. P-values described in Additional files 2 and 10.

A first examination showed that CFinder could extract modules that map to different sub-units of large complexes. For instance, the *RNA polymerase *complex (Int-7 High confidence) which is located in the nucleus, has DNA-directed RNA polymerase activity, and is composed of *RNA polymerase I *for the synthesis of rRNA 28S, 5.8S e 18S, then *RNA polymerase II *for the synthesis of mRNA, and *RNA polymerase III *for the synthesis of tRNA, rRNA 5S, snRNA and scRNA. Last, the *Proteasome *complex (Int-18 Medium confidence), with proteolitic activity, is composed of one central unit 20S and two caps 19 S.

Instead, MCODE could extract modules that map to specific complexes such as the *Mediator *complex Lit-11 Low confidence), a multiprotein complex that functions as a transcriptional co-activator, or the *Mrp *complex (Lit-4 High confidence), a ribonucleoprotein complex that performs the first cleavage in rRNA transcript processing, and is also involved in mitochondrial RNA processing.

#### Precision versus Recall

While precision addresses exactness or accuracy, recall addresses completeness. We computed these measures twice, and for each predicted module, in order to emphasize modules showing sufficient density in terms of proteins. Notably, this has been done for all the modules selected according to confidence scoring within the specific interactome classes. The values reported in Table 7 have been calculated for the Lit-PI, the Int-PI and the Ortho-PI datasets, according to CFinder and MCODE.

Therefore, we represented the protein complexes showing a good match module-wise, which thus involved the examination of each interactome class. In particular, the complexes could be deemed well matched when at least a 0.5 frequency matching threshold was reached (i.e. at least fifty per cent of the proteins in the known complex were matched by the proteins in the predicted module).

Then, precision and recall were computed protein-wise by each interactome class, but the *tp *were proteins in the predicted modules matching proteins in MIPS complexes, the *fp *were proteins in the predicted module not matching proteins in MIPS complexes, and the *fn *were proteins in MIPS complexes with no reference in the predicted module.

#### Permanent versus Transient Modules

Next, we have considered classification of the extracted modules in both permanent and transient protein associations, and have then established the protein pathway context within which this dichotomy occurs for some of the cases under study.

Literature on yeast protein complexes ([56,57]) has shown relationship of protein-protein interactions with mRNA expression levels, with consequent characterization of permanent versus transient complexes. We have referred to permanent and transient complexes by our approach too: while the former are maintained throughout the cell cycle and most conditions, the latter do not consistently maintain their interactions, and are involved in part of the cell cycle or in just some cellular states.

In our examples, it is through the impact of each method at the three confidence levels and across the various data types that we have assessed the co-expression scores to distinguish between complexes. We measured the correlation mean values for the mapped modules, which were also annotated in KEGG.

Similarly to the results obtained for yeast, the modules matching the *Proteasome *and *Ribosome *complexes (with high correlation scores) can be identified as permanent complexes, and the modules matching the *Transcription factors *complexes (with low correlation scores) as transient complexes.

Regarding the methods, CFinder is more redundant than MCODE (it maps three *RNA polymerase*, three *Proteasome*, and two *Transcrption factors*), but they include permanent and transient modules. Correspondingly, a combination of dynamic and static components had been observed in the sixty protein modules found to vary with the yeast cell cycle in [57].

In particular, for the *RNA polymerase *complex we have observed two different expression scores: in one case we found a high score in favor of a permanent complex, while in two other cases the scores were low, in favor of transient complexes.

#### A Look at Protein Pathways

Modules can be more or less cohesive, but their relevance must be seen in relation to the degree at which they communicate, particularly at the pathway scale. We have compared our confidence scoring approach with that of STRING [58], which investigates functional protein association networks. Thus, we have queried STRING with the extracted modules to assess the quality of validation relatively to our approach.

In particular, the concept of evidence in STRING implies the recourse to many different biological sources to validate the interactions, similarly to what we have done with disaggregation. Therefore, a few evidence categories (neighborhood, experimental, text mining) have been comparatively assessed in relation to our disaggregated data.

Pathway information has been evaluated too, from the database evidence in STRING and from our own integrative analysis. We cast our confidence levels within the STRING pathway framework based on as coring procedure which assigns a predicted value to each possible link between enzymes in metabolic maps from the KEGG db.

**Figure 5:**
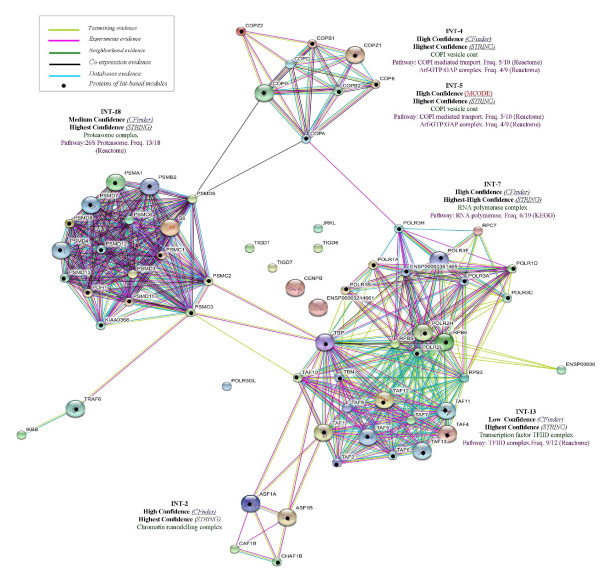
**Int-PI evidences**. Text mining, High-throughput, Co-expression, Neighborhood and Database STRING evidences compared to Int-PI based modules.

**Figure 6:**
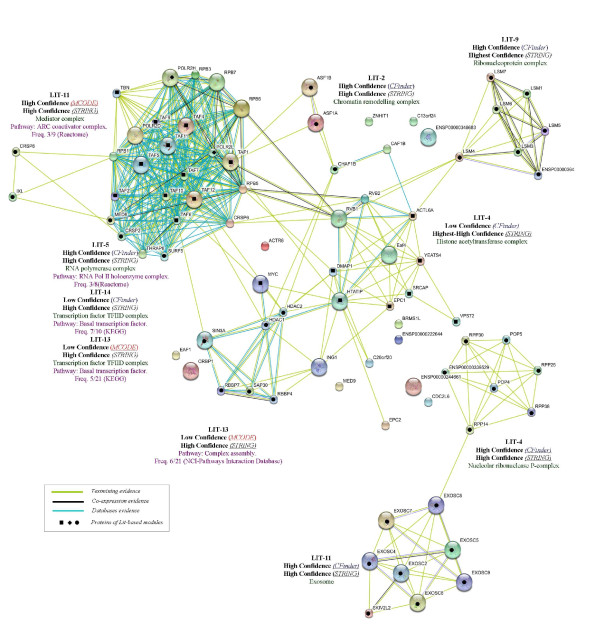
**Lit-PI evidences**. Text mining, Co-expression and Database STRING evidences compared to Lit-PI based modules.

Comparisons for two datasets are reported in Figure 5 and Figure 6, where Int-PI and Lit-PI are analyzed by multiple evidences. We have also introduced, for each case, pathway information through database evidence. The corresponding plots with reduced evidences have also been reported (supporting information in the Appendix). As a benchmark for comparison, we have marked with bullets the proteins identified by our approach in each STRING-based module. We emphasize three main aspects of our outcomes:

1. Int-PI and Lit-PI datasets. We found that some modules, such as Int-13 and Lit-14 from CFinder and Lit-13 from MCODE, and all referring to *Transcription Factors*, together with Int-2 and Lit-2 from CFinder related to *Chromatin remodelling*, were in agreement between the approaches. Some modules were uniquely validated in the Lit dataset, such as Lit-4 (*histone acetyltransferase *and *nucleolar ribonuclease*) from CFinder, Lit-9 (*ribonucleoprotein*) from CFinder, Lit-11 (*exosome *and *mediator*), from CFinder and MCODE, respectively. We found from Int two distinct modules, Int-18 (*proteasome*) from CFinder, and Int-4 (*COPI vesicle*) from CFinder that corresponds to Int-5 from MCODE. Last, we noticed that the RNA Polymerase complex is richer in terms of evidences in the Int-PI dataset.

2. Incorporating pathways. When the database evidence is included, *Transcription Factors *in both the Int-PI and the Lit-PI datasets resulted denser and linked to RNA Polymerase. Also, we found that the cross-links between some modules (Lit-2 and Lit-4 from CFinder, and Lit-13 from MCODE) related to *Chromatin Remodelling *were reinforced. Last, the analysis of the Int-PI dataset confirmed these outcomes.

3. Matched annotation. Overall, we have found only to a marginal extent the presence of isolated nodes that remained non-annotated, while most modules (with proteins indicated by bullet points) matched very well with STRING evidences. By comparing our confidence scoring scheme with STRING confidence levels, we noticed that for the Int-PI dataset there is a strong match, with just one case of mismatch for Int-13 from CFinder. For the Lit-PI dataset we found the same strong match, and just two cases of mismatch for Lit-13 from MCODE, and Lit-14 from CFinder.

### Scoring Calibration

We verified (see Additional file 1) that modules ranking at the top 5 positions confirmed after calibration their pre-assigned confidence levels (overall, 18 rank places are taken by High Confidence modules, 9 by Low Confidence modules and only 3 by Medium Confidence modules, but the latter were marginally present and even missing in MCODE). We could thus conclude that the initial scoring system was quite robust, but could be refined by calibration through multiple validations. With regard to the interactome classes, we observed that the top 5 ranks involved equally all the classes (10 each), and with marginal prevalence for Int-PI in CFinder and for Ortho-PI in MCODE.

We found that the best overlap scores were uniformly distributed among modules according to their pre-assigned confidence levels, while interactome classes had an impact depending on the method. On average, for CFinder the Lit-PI modules scored 0.7 - 0.8 (computed as min-max indexed values), while the other two classes scored 0.3 - 0.5, and for MCODE the Int-PI scored around 0.9 (but only one module was annotated) and the other two classes scored 0.3 - 0.5, with Lit-PI closer to the higher limit. Overall, we found evidence confirming the goodness of our initially assigned confidence levels, and additionally observed method's sensitivity when averaging overlap scores over interactome classes.

In examining the FDR-corrected p-values in pathways, we noticed that just a few cases could be quantified, while we observed that the participation degree of each module to intra-modular or inter-modular dynamics was clearly induced by the methods. CFinder determined evidence of cross-talks in several cases and with both dense and sparse modules, but regardless the interactome class or the pre-assigned confidence levels. MCODE instead showed only for the Lit-PI class evidence of inter-modularity through dense modules, while Ortho-PI appeared just cohesive.

The calibration approach applied to the initial scoring system provided therefore additional evidence by looking at GO p-values, but also at overlap scores and pathway analysis, which were not previously included. In particular, the analysis across the interactome classes revealed dependence on methods of the overlap scores (where the Lit-PI overperforms in CFinder), and of both intra-modular and inter-modular dynamics (where the Lit-PI overperforms in MCODE, this time). It is the sparsity degreee of modular maps produced by the methods which suggests how to interpret these findings. MCODE allows for a coarse simplified analysis of cross-talks compared to CFinder, and emphasizes the relevance of interactome classes in particular. Instead, the presence of high overlapping degree in CFinder appears to mask the differential impact of classes. Thus, based on the resolution spectrum allowed by the modularity method and depending on the induced sparsity of the map, the interactome classes may have a relevant or a marginal role.

Confidence schemes (see [59-61]) usually address the problem of selecting reliable interactions by intersecting multiple high-throughput data (omic) sets. Weights can be assigned to the interactions, and they represent confidence levels that rank the proteins by their likelihood of belonging to a certain annotated complex or functional module. Estimates of the chance that a protein-target set association exists are in general obtained as the fraction of sampled networks that include a path connecting the candidate protein to the set.

In general, the confidence level thus reflects a belief about the likelihood of a biological module. In particular, determining confidence for protein interactions depends on assigning a reliability measure to the observed measurements, or to predictions. Thus, confidence scores are formulated according to the type of experiment, or according to a combination of features like functional similarity, expression correlation, co-essentiality, over which the predictions have been formed.

Our scoring approach is semi-quantitative, and calibrates confidence measures obtained by thresholding through validations conducted across multiple biological sources. As a result of filtering interactions and modules by confidence levels, the heterogeneity of modularity maps that depends on both methods and interactome datasets may be reduced according to a better control of the uncertainty levels. For instance, the suggested scoring yields an empty MCODE Medium Confidence module set, while the noisier HTP modules are missing.

## Conclusions

We have applied popular graph mining methods, in particular CFinder and MCODE, to different classes of human protein interactomes, and extensively evaluated their induced modularity maps. Both MCODE and CFinder are deterministic methods based on node connectivity and cliquishness. We have then validated the extracted modules by integrating the annotation of known MIPS complexes, GO categories and pathways. The proposed multilayer analysis has thus involved protein pathway explorations through modules with assigned confidence measures.

We have achieved three main results. First, by verifying that interactome disaggregation reveals useful information on modularity, we emphasized the fact that in some cases annotation can be performed with no reference to the integrated dataset, which is usually the starting point of most analysis.

Second, our approach defines a scoring mechanism from protein interactions to modules which might lead to a novel network design because the scoring is usually performed at the beginning, for instance at the experimental phase, and not a posteriori (during in silico biological validation) as we suggest. Notably, this leads to a network calibration strategy aimed to possibly shift an un weighted network to a weighted one through biological validation evidences that are incrementally evaluated and combined.

Assigning confidence scores computationally and not at the experimental stage allows the application of a large variety of possible schemes. Here, we pursued a simple data-driven approach corroborated by multiple validations instead of a more complex model-oriented approach. Our choice offers the advantage that it does not assume a specified model for the data with its implicit risks, but can be adapted to the quality of interaction data derived from experiments, databases or computational predictions. This simple procedure turned out to be an effective strategy that might help to improve the reliability of PPIN.

Last, as we have implemented an approach for combining protein interactions with gene expressions, we have found modules which are more consistent than those obtained by using only the topology of the network itself, and then refined their validation with reference to permanent versus transient modules, together with other proposals (e.g. overlap scores, pathway enrichment). Future work is going to be devoted to further calibration of the thresholding step in a data-adaptive way, and to tune it to a better elucidation of the interaction-expression interplay.

Regarding the initial questions, the main issue is about the influence of data integration and/or disaggregation on modules. From a quantitative standpoint, we demonstrated that protein complex detection benefits from interactome disaggregation. We have reported evidence of well-defined modules which have been distinctly detected within disaggregated datasets, and have compared our approach with STRING through its variety of evidences accounting for different information sources.

We presented examples where our class separation was matched by comparable evidences leading thus to similar validation performance under both the Int-PI and Lit-PI datasets. Inference on the qualitative characterization of the various different modules indicates replication effects in CFinder, i.e. two disaggregated datasets appear similarly informative, and nesting effects in MCODE, i.e. similar biological evidence persists at both the integrated and the disaggregated levels. This outcome might depend only in part on the number of modules that each method is producing; thus, their structural features (redundancy versus sparsity) seem to reasonably explain the different performances.

Another question referred to modularity dependence on algorithms. Overall, we noted that MCODE produced less modules than CFinder in every dataset which was considered. In particular, MCODE has generated only a few modules (from 11 modules in HTP-PI to 141 modules in Ortho-PI), thus tending to perform sub-optimally with regard to the global resolution at which the interaction dynamics occur. Instead, the module overlapping evidence by CFinder has been more emphasized (from 61 modules in HTP-PI to 1096 modules in Int-PI), despite substantial module specificity in the disaggregated interactomes. The related distributional aspects have been also examined by inspection of the resolution of module sizes and their associated power laws.

Then, we have explored module overlapping and separation effects, and their data-driven or method-driven nature. Together with MCODE and CFinder, which effectively represent coarse-to-fine coverage of modularity maps, we have implemented a deterministic method (MaxMod) and a stochastic algorithm (Walktrap) to allow for variation of the resolution spectrum. The results ranged from a minimum of 129 modules by MaxMod and 151 modules by Walktrap for HTP-PI, to a maximum of 211 modules by MaxMod for Ortho-PI and 314 modules by Walktrap for Int-PI). Overall, the insertion of both greedy and stochastic learning features allowed a reduction of the resolution distance exhibited by the previous methods, but performed (especially MaxMod) more similarly to MCODE, thus still inducing sparsity to a certain extent.

Overall, despite some kind of sparsity-redundancy trade-off that is inherent to the module extraction method, protein complexes can be distinctly characterized based on disaggregated interactomes. A closer look at the interactomes is thus recommended, as protein associations may find stronger or weaker justification in relation to the specific sources used to measure them and build the datasets. The proposed multilayer approach offers insights on how the specific interactome datasets may determine the performance of modularity detection algorithms, and suggests strategies to refine their biological validation.

## List of abbreviations

We used the following abbreviations: PPIN: protein-protein interaction networks; MAXMOD: maximization of modularity; LIT-PI: literature-based interactome; ORTHO-PI: orthology-based interactome; HTP-PI: high-throughput interactome; INT-PI: integrated interactome; RW: random walk.

## Authors' contributions

EM conducted method and algorithm implementation, with data analysis . AT performed biological validation, module characterization and graphical representation. GC and MF designed variable selection method based on gene co-expression and built UniHi db. EC conceived confidence scoring approach, methodological analysis, organized and wrote the paper. Every author contributed to writing parts of the manuscript, and then approved the final draft.

## Appendix

### Materials and methods

#### Residual Modules

Table 8 and Table 9 report results of GO-based annotation for respectively CFinder and MCODE modules whose values have not passed our confidence scoring thresholds.

**Table 8:** GO-based annotation of residual CFinder modules

	n	n.ex	ex.mean	cor.mean	mf.mean	bp.mean	cc.mean
Int-1	1496	1348	8.73	0.13	NA	NA	NA
Int-5	7	4	10.71	0.57	0.31	0.35	0.42
Int-6	8	5	8.39	0.39	0.34	0.32	**0.54**
Int-8	8	8	9.5	0.44	**0.5**	0.46	**0.62**
Int-9	8	6	8.68	0.49	0.27	0.46	**0.81**
Int-10	12	10	8.64	0.41	0.39	0.49	**0.61**
Int-11	9	9	9.08	0.41	0.42	0.26	**0.64**
Int-12	11	10	8.84	0.26	0.35	0.36	**0.5**
Int-14	14	9	8.76	0.4	0.37	0.2	**0.67**
Int-15	15	9	8.42	0.46	**0.55**	0.23	**0.8**
Int-16	14	8	8.42	0.46	0.43	0.21	**0.76**
Int-17	28	13	12.06	0.6	0.46	**0.56**	0.49
Int-19	21	9	10.03	0.28	0.4	0.48	0.47
Int-20	18	6	9.07	0.36	0.47	0.34	**0.59**
Int-21	22	10	9.5	0.12	0.39	0.48	0.47

Lit-1	1141	1081	8.6	0.12	NA	NA	NA
Lit-3	6	6	9.37	0.15	**0.69**	0.15	0.37
Lit-6	6	6	8.21	0.63	**0.55**	0.44	**0.56**
Lit-7	8	5	8.39	0.39	0.34	0.32	**0.54**
Lit-8	7	5	8.14	0.18	**0.76**	0.19	0.42
Lit-10	10	10	9.32	0.46	0.35	0.15	**0.77**
Lit-12	9	9	8.64	0.11	0.22	0.27	0.38
Lit-13	11	10	8.84	0.26	0.35	0.36	**0.5**

Ortho-1	7	2	8.6	0.5	0.41	0.19	**0.57**
Ortho-2	4	3	8.34	0.37	**0.62**	0.22	**0.55**
Ortho-3	4	3	8.58	-0.18	**0.54**	0.35	**0.7**
Ortho-4	4	4	8.65	0.69	0.44	0.45	0.48
Ortho-5	14	10	8.86	0.1	0.34	0.24	0.39
Ortho-6	7	4	10.71	0.57	0.31	0.35	0.42
Ortho-9	8	8	9.5	0.44	**0.5**	0.46	**0.62**
Ortho-10	8	6	8.68	0.49	0.27	0.46	**0.81**
Ortho-11	12	10	8.64	0.41	0.39	0.49	**0.61**
Ortho-13	58	29	10.81	0.42	0.38	0.47	0.47
Ortho-14	24	7	9.21	0.39	0.45	0.43	**0.61**
Ortho-15	14	9	8.76	0.4	0.37	0.2	**0.67**
Ortho-16	39	22	10.67	0.35	0.43	0.49	0.49
Ortho-17	15	9	8.42	0.46	**0.55**	0.23	**0.8**
Ortho-19	21	9	10.03	0.28	0.4	0.48	0.47
Ortho-20	18	6	9.07	0.36	0.47	0.34	**0.59**
Ortho-21	22	10	9.5	0.12	0.39	0.48	0.47

HTP-1	5	5	12.73	0.21	**0.55**	0.2	**0.56**
HTP-2	3	3	7.79	0.06	**0.78**	0.24	0.39
HTP-3	3	2	7.33	0.07	0.46	0.1	**1**
HTP-4	145	108	8.33	0.06	NA	NA	NA
HTP-5	3	2	7.97	0.05	0.44	0.05	0.29
HTP-6	163	120	8.99	0.15	NA	NA	NA
HTP-7	44	36	8.96	0.16	0.34	0.17	0.44
HTP-8	7	5	8.38	-0.06	0.31	0.12	0.31
HTP-9	5	5	9.16	0.03	**0.78**	0.36	**0.66**

GO-based annotation results for CFinder modules. Values in bold font are above the 0.5 threshold. The listed groups have passed less than three thresholds, and thus received a low score.

**Table 9:** GO-based annotation of residual MCODE modules

	n	n.ex	ex.mean	cor.mean	mf.mean	bp.mean	cc.mean
Int-1	14	13	7.37	0.1	0.49	0.44	**0.5**
Int-2	9	7	7.83	-0.02	0.27	0.24	**0.52**
Int-3	13	9	8.66	0.33	0.47	0.41	**0.66**
Int-4	13	12	8.29	0.06	0.4	0.44	**0.58**
Int-6	75	68	8.46	0.2	0.24	0.18	0.4
Int-7	58	43	8.3	0.19	0.26	0.16	0.38
Int-8	47	39	9.1	0.23	0.25	0.17	0.44
Int-9	74	70	9.44	0.28	0.27	0.24	0.43
Int-10	10	6	10.28	0.77	**0.65**	0.43	**0.77**
Int-11	29	27	9.12	0.4	0.36	0.33	**0.63**
Int-12	32	28	8.72	0.35	0.31	0.41	**0.61**
Int-13	79	46	9.99	0.49	0.36	0.31	0.48
Int-14	29	12	9.77	0.23	0.37	0.44	0.43

Lit-1	5	5	7.87	0.02	0.53	0.06	0.46
Lit-2	7	7	8.47	-0.01	0.44	0.32	**0.74**
Lit-3	145	139	8.42	0.06	0.25	0.15	0.35
Lit-5	5	2	8.69	0.16	0.3	0.16	**0.61**
Lit-6	144	140	8.69	0.1	0.26	0.19	0.41
Lit-7	21	21	7.98	0.05	0.33	0.19	0.39
Lit-8	98	96	8.72	0.16	0.24	0.24	0.41
Lit-9	15	15	8.67	0.15	0.26	0.2	0.45
Lit-10	128	122	8.62	0.21	0.28	0.16	0.42
Lit-12	40	39	8.79	0.23	0.29	0.28	**0.51**

Ortho-1	6	3	11.12	0.67	0.32	0.37	0.39
Ortho-3	12	8	8.65	0.32	0.45	0.37	**0.66**
Ortho-6	13	6	8.38	0.24	0.28	0.2	**0.53**
Ortho-7	10	10	9.16	0.12	0.44	0.21	**0.68**
Ortho-8	23	20	10.08	0.51	0.35	0.33	**0.56**
Ortho-9	10	6	10.28	0.77	0.65	0.43	**0.77**
Ortho-11	44	31	10.58	0.53	0.35	0.43	0.48
Ortho-12	35	15	8.81	0.45	0.44	0.28	**0.64**
Ortho-13	35	15	8.81	0.45	0.44	0.28	**0.64**
Ortho-14	29	12	9.77	0.23	0.37	0.44	0.43

HTP-1	67	56	8.74	0.12	0.3	0.16	0.41
HTP-2	17	15	9.23	0.17	0.45	0.16	0.44
HTP-3	20	18	9.06	0.08	0.45	0.28	**0.5**
HTP-4	17	15	8.83	0.14	0.38	0.16	0.37

GO-based annotation results for MCODE modules. Values in bold font are above the 0.5 threshold. The listed groups have not passed at least the two required GO thresholds, and thus received a low score.

#### MIPS Annotation

**Figure 7:**
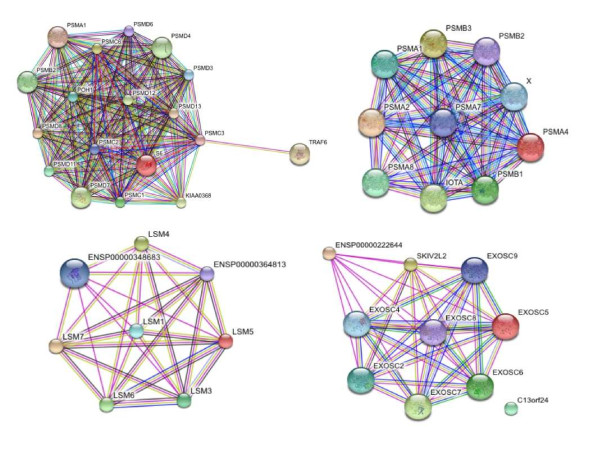
**STRING-based modules annotation**. *Top-left plot*: output from STRING of the Int-18 Medium confidence CFinder module query. *Top-right plot*: output from STRING of the Ortho-12 High confidence CFinder module query. *Bottom-left plot*: output from STRING of the Lit-9 High confidence CFinder module query. ENSP00000348683 is the SMN1 protein. *Bottom-right plot*: output from STRING of the Lit-11 High confidence CFinder module query. ENSP00000222644 is the MPP6 protein.

We observed that CFinder could extract modules which match well with multiple MIPS complexes, and in some cases also functionally related to other proteins (which we found with STRING, a tool based on different type of evidences with a corresponding score), or instead with relatively poor match with MIPS. This is the case of proteins belonging to the Int-18 Medium confidence module, which map to the *Proteasome *complex. However, the protein TRAF6 (TNF receptor-associated factor 6) that maps to small MIPS complexes, do not belong to the Proteasome. Instead, from STRING we have observed (Figure 7, top-left plot) a functional relation of TRAF6 with PSMC3 of the Proteasome complex.

An example refers to the Ortho 12 High confidence module that has a match with the *Proteasome *complex, but also with the PSMA8 protein which is not present in MIPS. When we looked at STRING we found that this protein too is functionally related (Figure 7, top-right plot). Furthermore, an example is provided by the Lit-9 High confidence module, which has a match with the *LSM1-7 *complex (involved in mRNA processing). The SMN1 protein (*Survival motor neuron*) associated to the SMN complex (also involved in mRNA processing), is linked in STRING with other proteins of the *LSM *complex (Figure 7, bottom-left plot). The last example consists in the Lit-11 High confidence module that has a match with the *Exosome *complex, but not through a protein, MPP6, which instead is associated to the *Exosome *by STRING (Figure 7, bottom-right plot).

#### Precision and Recall

While precision indicates for a certain group of elements the number of true positives (i.e. correctly labelled as belonging to the group) divided by the total number of elements labelled as belonging to the group (i.e. the sum of true positives and false positives, where the latter are incorrectly labelled as belonging to the group), recall is defined as the number of true positives divided by the total number of elements that actually belong to the group (i.e. the sum of true positives and false negatives, where the latter are not labelled as belonging to that group, but wrongly though).

**Figure 8:**
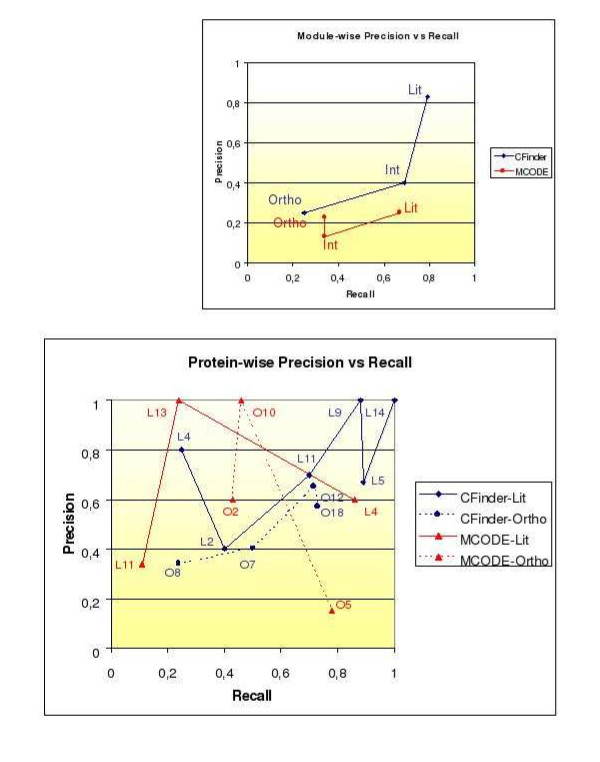
**Precision vs Recall Patterns**. Lines and symbols according to methods, datasets and confidence levels. *Blue line*: CFinder (Lit): L5 = Lit-5 High; L2 = Lit-2 High; L11 = Lit-11 High; L14 = Lit-14 Low; L9 = Lit-9 High. *Red line*: MCODE (Lit): L11 = Lit-11 Low; L4 = Lit-4 High; L13 = Lit-13 Low. *Red dots*: MCODE (Ortho): O5 = Ortho-5 High; O2 = Ortho-2 Low; O10 = Ortho-10 Low. *Blue dots*: CFinder (Ortho): O8 = Ortho-8 High; O7 = Ortho-7 Low; O12 = Ortho-12 High; O18 = Ortho-18 Medium.

Figure 8 shows "Precision vs Recall" patterns obtained at both levels of our analysis. The top plot shows method-driven Precision versus Recall patterns module-wise and across datasets. The bottom plot shows method-driven precision vs recall patterns protein-wise and across the modules of two disaggregated datasets.

For each predicted module, after first looking at the best annotation by GO cellular component according to the computed *p*-values, we have considered the modules that matched MIPS complexes relatively to the GO annotation. For each matched complex we have estimated the corresponding frequency (a complete list of MIPS-matched complexes and related frequencies is provided in the Additional file 5).

In order to estimate precision and recall of Eq. (2), we took the MIPS-matched complex with the best frequency (see the Additional file 6). Instead, in order to estimate precision' and recall' (as from Eq. (3) formulas), we kept only the frequencies ≥0.5. Therefore, *P *was calculated as the ratio between thefrequencies ≥0.5 over the number of all the detected MIPS complexes. For the estimation of *R*, where *MC *was calculated as the mean of the frequencies involved in the same module, the ratio has involved the mean frequencies ≥0.5 over the number of predicted modules.

Note that the Int-PI dataset referred to MCODE, and reported in Figure 8 like the other datasets, has requested a less stringent selection in order to be considered (originally, only one module, Int-5, had been selected). Instead, the three modules (Int-3, Int-10, Int-14) that have been newly introduced were not in the initial selection. Thus, we relaxed the selection criterion by accepting lower confidence to include more examples from MCODE. However, the frequencies related to Int-3 and Int 14 are low because these modules are mapped against big complexes, which in turn emphasizes the differential performance of MCODE in comparison to CFinder.

#### Data Sources

Through UniHI we have access to more than 253, 000 distinct interactions between over 22,300 unique human proteins. UniHI is a comprehensive database of both computational and experimental human protein interactions aimed to integrate various possible protein maps and publicly accessible at http://www.unihi.org. From the sources available within UniHI, we have considered the following disaggregated datasets to specify our interactome classes: HTP-PI, which is a rich yeast two hybrid (Y2H) network; Ortho-PI, which consists of thousands of interactions that are computationally predicted from experimentally measured interactions in lower model organisms such as yeast, fly and worm; Lit-PI, which represents literature-curated binary interactions built from BIND, DIP, BIOGRID, HPRD-Binary, and INTACT db sources. Finally, we have considered a fusion of all the data from the disaggregated interactome, which we call the Int-PI.

We point out that the overlap between the Y2 H human data set and literature-curated interactions is limited to only 10% [11], which represents a weak intersection due to the superior accuracy achieved by the latter approach. However, recent work [62] has cast doubts over curation's accuracy, thus calling for re-curation strategies.

#### Modules extraction techniques

Algorithms for graph mining and cluster detection in networks are mainly based on network flow and minimum cut theory ([63,64]), and also on spectral clustering [65]. We have focused on two popular deterministic methods, MCODE and CFinder, in comparison with other algorithms allowing for different search strategies (i.e. MaxMod and RW).

MCODE stands for Molecular Complex Detection, and discovers densely connected regions that may be associated with molecular complexes. It is based on a node-weighting concept that utilizes the clustering coefficient to measure the cliquishness of the neighborhood of each node, and computes fully connected subgraphs of a given minimal degree *k*, called *k*-cores.

Thus, for each given *k *each node in a *k*-core has connectivity degree greater or equal to *k*; thus, *k*-cores potentially embed (*k *+ *p*)-cores, where *p *is a positive integer. The MCODE algorithm scores and ranks each resulting complex according to both its density and its size.

CFinder detects overlapping dense groups of nodes in un weighted undirected networks, or *k*-clique "communities", based on the *Clique Percolation Method*, and by the analysis of a so-called clique-clique overlap matrix.

A *k*-clique community is the union of smaller complete fully connected subgraphs that share nodes: thus a group of clusters composed by *k *nodes fully connected to each other in the same cluster, but not necessarily with many others in the community.

We note that *k*-cores and *k*-clique communities differ in some sense: in the former case each node is connected with at least k other nodes (here *k *refers to the connectivity degree), while this is not true in the latter case, where *k *is the number of elements in each clique of the community.

Consequently, both MCODE and CFinder are essentially meant to find highly connected subnetworks, but while MCODE cores are fully connected (each node connects with each other), each node in CFinder' smodules is not required to be connected to all other nodes.

MCODE and CFinder present a few differences, which may affect the biological relevance of findings. The most striking aspect is that CFinder locates overlapping modules: a given node can be a member of several different communities at the same time, and communities can overlap with each other by sharing nodes. This overlap occurs also with MCODE because of its nested structure visible through the *k *sequence, but good layer separation can control the induced redundancy. Overlaps reflect an important property, as the module cross-links can emphasize a particular interface role for proteins with regard to multiple biological processes.

#### Method parameterization

Both MCODE and CFinder require the tuning of some parameters which are described in the corresponding (freely available) software packages. In MCODE, a score is associated to each module by the ratio between the number of interactions and that of proteins in the module. In Additional file 7, the structure of cores found for *k *= 2 and for each dataset are shown as an example. The user-controlled parameters for implementing and running the algorithm are set in our numerical experiments as follows:

1. *FLUFF = false*, was chosen to emphasize the best possible cluster separation degree aimed to identify as many unique modules as possible, and to control the possible redundancy induced by the nest-based structure;

2. *HAIRCUT = true*, was chosen to remove all single (orphan) nodes and allow for at least 2-core clusters;

3. *Node Score = 0.2*, was kept at default value not to have too small modules as an outcome;

4. *Max Depth = 100*, was chosen to avoid the presence of very small modules.

The rationale behind our selection of clique sizes and their modules considers one factor: we try to avoid the redundancy inherent to the nested structure (i.e. a protein can belong to different clique sizes *k *because associated in multiple ways, but every time which is present in a certain *k_i _*results also present in all *k*_*≤i *_by construction). Thus, while we keep track of the sequence of *k *values, we consider all modules produced by the minimal *k*, say *k *= 2. Consequently, we can establish each module's assignment according to specific connectivity degrees, and label it according to coreness levels from which biological associations can be investigated.

In CFinder, for each interactome class (INT, LIT, ORTHO, HTP) a list of entries for various *k *(clique size values for the communities) is reported in the Additional file 8, and refers to the number of retrieved communities, the ordinal number of selected communities, and the given ID.

As the original number of communities is quite high, some reduction steps have been undertaken. A first forward reduction step has involved the consideration of only *k *≥ 4 (thus skipping the giant components, while still gaining useful information for sparse networks), and in each case only a representative sub-set of five communities (i.e. a sub-sample including mid-sized communities, instead of too small or big ones) of the output file was retained (except for HTP, where they are originally just a few).

Then, the second reduction step has involved a backward selection to limit redundancy as follows: starting from the biggest *k*, we have chosen communities for each *k *in an exclusive manner, i.e. only if they had not been selected yet, thus avoiding duplicates and too big clusters (say more than 200 elements). After the identification of the communities at the high-size k-clique level, more communities were selected and added incrementally from the smaller k-cliques so to introduce proteins not yet visited.

The rationale of the above strategy is that we sought a rapid detection of communities for each value of *k*. Thus, we sequentially added communities with decreasing k-clique size, while keeping track of the previous community structure, and therefore limited both the redundancy and the overlapping effects. We looked at shared communities between the new k-cliques and those already considered. We allowed for all possible *k *apart from *k *= 2 (pairs of nodes connected by single links and 1-cliques are single nodes). Note in Table 10 that the symbol "-" indicates that the communities of the corresponding *k *are almost totally overlapping with the communities of *k *+ 1, thus yielding the same groups as before.

**Table 10:** CFinder communities

	Int-PI	Lit-PI	Ortho-PI	HTP-PI
k = 3	448	364	183	53
k = 4	328	248	71	8
k = 5	134	88	44	-
k = 6	69	40	27	-
k = 7	42	21	20	-
k = 8	24	10	10	-
k = 9	11	4	6	-
k = 10	7	2	4	-
k = 11	7	2	5	-
k = 12	5	-	5	-
k = 13	6	-	6	-
k = 14	7	-	7	-
k = 15	4	-	4	-
k = 16	3	-	3	-
k = 17	2	-	2	-
TOT	1096	779	397	61

CFinder communities across datasets for varying k.

For MaxMod and Walktrap methods, parameterization is less requiring. While for the former method we just counted the number of modules each time a maximum for the modularity function was obtained, for the latter method we kept the length of the RW to default values, thus allowing for module merging to be as much informative as possible (i.e. neither too stringent nor too conservative in considering the most relevant modules).

Recently, the approaches proposed in Newman [66,67] suggest that a graph should be split in a hierarchy of modules, for instance by successively removing links with large betweenness (or variants of it), where this property defines the number of shortest paths crossing a link. However, due to the slow convergence, these approaches have been found to be often unfeasible for large networks, as in our cases.

### Other Supporting Information

**Figure 9:**
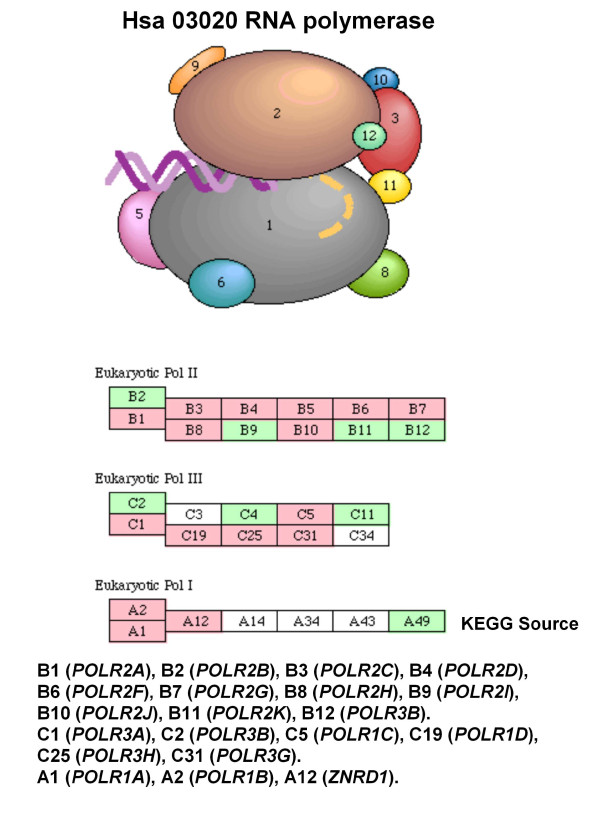
**Hsa 03020 RNA polymerase**. Listed in parenthesis the original labels for RNA polymerase. Int-7, Lit-5, Ortho-8 are CFinder High Confidence modules which map Hsa 03020 RNA polymerase.

**Figure 10:**
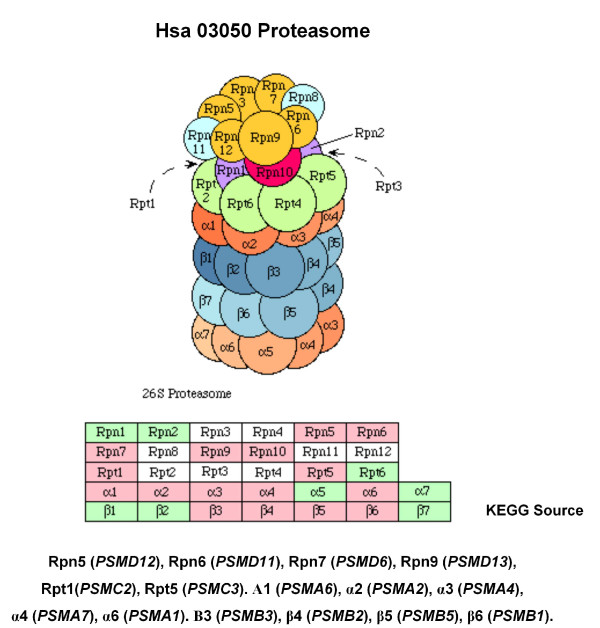
**Hsa 03050 Proteasome**. Listed in parenthesis the original labels for Proteasome. Ortho-12 is CFinder High Confidence module, while Int-18 and Ortho-18 are CFinder Medium Confidence modules which map Hsa 03050 Proteasome.

**Figure 11:**
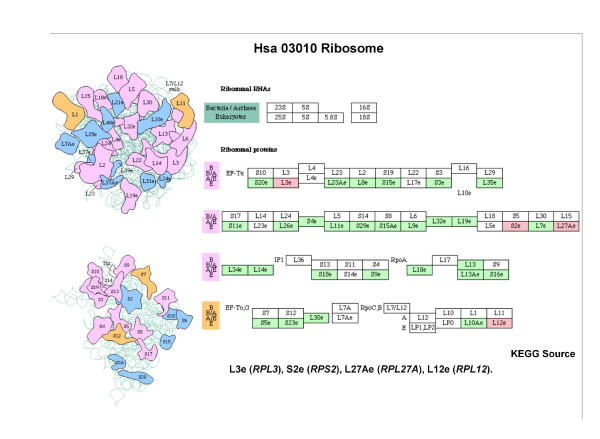
**Hsa 03010 Ribosome**. Listed in parenthesis the original labels for Ribosome. Ortho-5 is MCODE High Confidence module which maps Hsa 03010 Ribosome.

**Figure 12:**
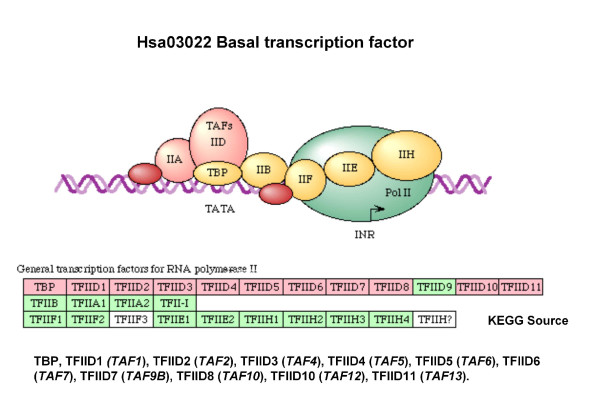
**Hsa 03022 Basal Transcription Factor**. Text mining, High-throughput, Co-expression and Neighborhood STRING evidences compared to Int-PI based modules.

**Figure 13:**
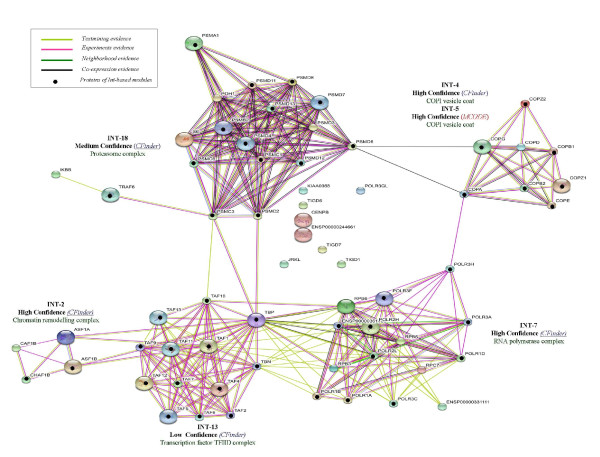
**Reduced Int-PI evidences**. Text mining, High-throughput, Co-expression and Neighborhood STRING evidences compared to Int-PI based modules.

**Figure 14:**
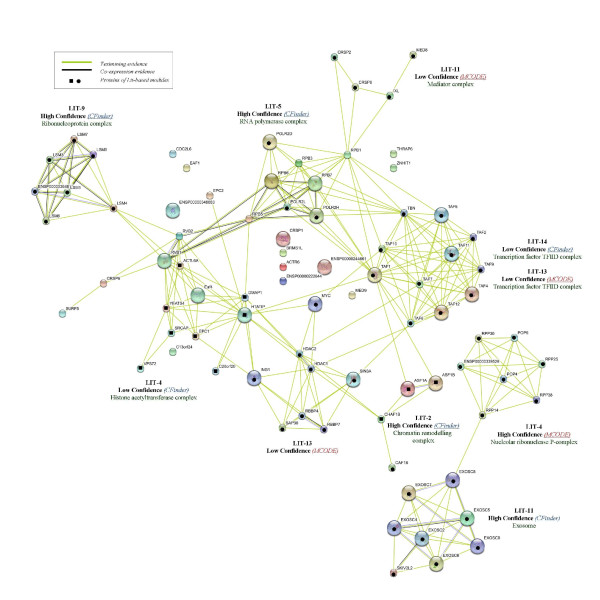
**Reduced Lit-PI evidences**. Text mining and Co-expression STRING evidences compared to Lit-PI based modules.

**Figure 15:**
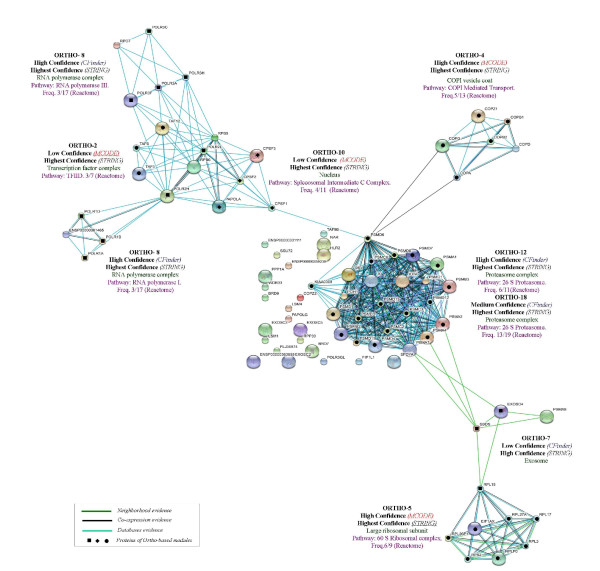
**Ortho-PI evidences**. Text mining, Co-expression and Database STRING evidences compared to Ortho-PI based modules.

**Figure 16:**
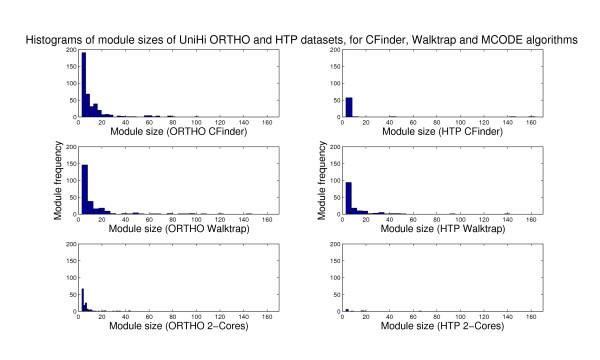
**Histograms of module sizes (Ortho-PI and HTP-PI)**. The three algorithms show distributional differences, particularly with HTP data. 2-cores appear with limited module detection power. From Matlab implementations.

**Figure 17:**
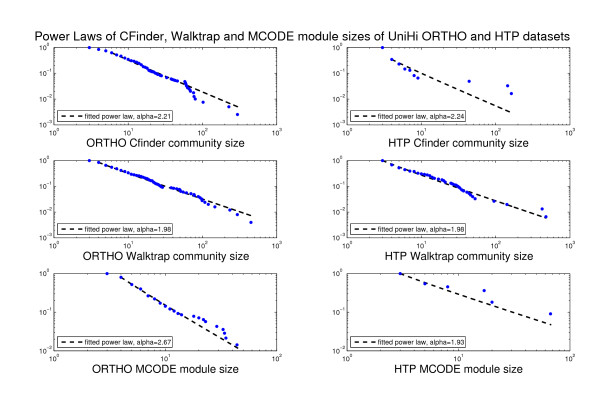
**Power Laws**. Power laws vary quite substantially for the two datasets, and depending on the method. From Matlab implementations.

**Figure 18:**
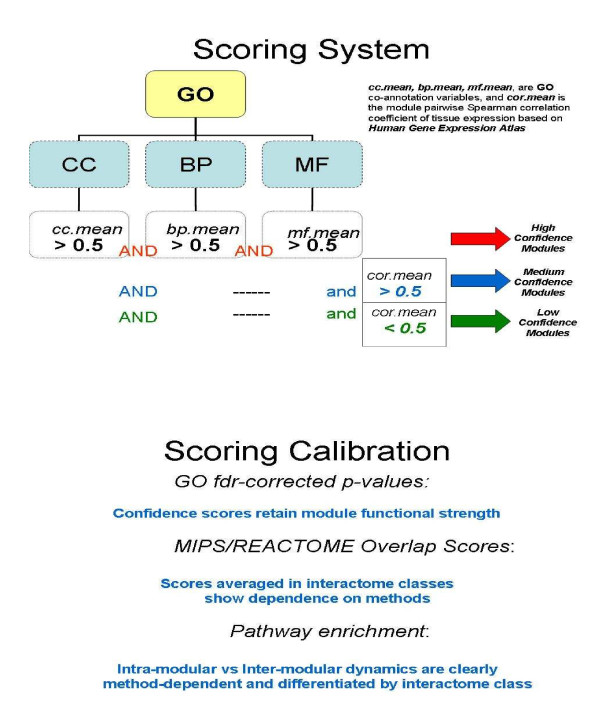
**Confidence Scoring System**. Confidence score assignment to modules according to thresholding performed over variables computed from the Human Gene Expression Atlas, and calibration by multiple validations.

Table 10 is for the CFinder community structure, and Table 11 reports on MaxMod and Walktrap convergence results. The Additional file 7: Supplemental Table S12 reports the core structure for *k *= 2 and each dataset, while the CFinder structure appears in the Additional file 8. Last, a protein labelling file for all datasets is provided in the Additional file 9: Supplemental Table S13. Figure 9, Figure 10, Figure 11 and Figure 12 refer to module-specific pathways. Then, Figure 13 and Figure 14 and Ortho-based comparisons with STRING in Figure 15 complete the examples reported in the main text relatively to confidence scoring methods. Figure 16 is about the distribution of module sizes for residual datasets, while Figure 17 shows the corresponding power laws. Figure 18 reports a sketch of our global confidence scoring system explained indetail in the main text. The Additional file 10 is for specifying the p-values computed in the examples, and the Additional file 11 reports the communities produced by the *Walktrap *algorithm.

**Table 11:** Modularity values

	MaxMod maximal modularity	Walktrap maximal modularity
**Int-PI**	0.4631	0.4176
**Lit-PI**	0.5074	0.4632
**Ortho-PI**	0.7181	0.6899
**HTP-PI**	0.6215	0.2600

Modularity values for MaxMod and Walktrap methods (details in Additional file 11).

## Acknowledgements

EM, AT and EC thank CRS4 and Sardegna Ricerche. The work by MF and GC was supported by the SFB 618 grant of the DFG. MF aknowledges support by the FCT (IBB/CBME, LA). Four anonymous referees contributed to substantial improvements of the paper. We thank our colleagues, Lance Liotta, Emanuel Petricoin, and Alejandro Giorgetti for comments, and Chiara Rotondi, who checked the general spelling.

## Supplementary Material

Additional file 1 **GOpvalues**. Semi-quantitative evaluation of GO-annotated modules with p-values. Click here for file

Additional file 2 **tableGOp-valuecomplete**. Complete list of GO-annotated modules. Click here for file

Additional file 3 **overlapscores**. Overlap scores computed for MCODE and CFinder modules. Click here for file

Additional file 4 **tablePathways**. Pathway annotation and evaluation for MCODE and CFinder modules. Click here for file

Additional file 5 **TablesModulewisePvsR**. P vs R scores computed for MOCDE and CFinder modules. Click here for file

Additional file 6 **TablesProteinwisePvsR**. P vs R scores computed for MOCDE and CFinder modules (disaggregated by interactome class). Click here for file

Additional file 7 **MCODEresults**. It contains four files: *MCODE2coresHTP.txt*, *MCODE2coresINT.txt*, *MCODE2coresLIT.txt*, *MCODE2coresORTHO.txt *that report core structure and parameters. Click here for file

Additional file 8 **CFinder-results**. It contains the complete set of communities retrieved by CFinder from the various interactome classes. Click here for file

Additional file 9 **Mapping**. Protein labels for all datasets. Click here for file

Additional file 10 **annotation-description**. Notes on the computed p-values. Click here for file

Additional file 11 **walktrap-results**. It contains the complete set of communities retrieved by *Walktrap *from the various interactome classes. Click here for file
