# Supplementary material for: Inferring modules from human protein interactome classes
Source: BMC Syst Biol. 2010 Jul 23;4:102. doi: 10.1186/1752-0509-4-102 (PMC2923113; doi:10.1186/1752-0509-4-102)
Supplement: Additional file 1 — GOpvalues. Semi-quantitative evaluation of GO-annotated modules with p-values. [file 1752-0509-4-102-S1.DOC]

**Semi-quantitative evaluation of GO-annotated modules with p-values (FDR-corrected) sorted by increasing order.**

Integrated with file ***tableGOp-value_complete.doc*** where full annotation has been reported*.*

| ***CFinder*** |  |  |  |
| --- | --- | --- | --- |
| **Rank** | **cc**  **(cellular component)** | **bp**  **(biological process)** | **mf**  **(molecular function)** |
| **1** | **LowC Int-13** | **MediumC Int-18** | **HighC Int-7** |
| **2** | **HighC Int-4** | **HighC Int-7** | *HighC Ortho-8* |
| **3** | **MediumC Int-18** | **LowC Int-13** | *HighC Ortho-12* |
| **4** | LowC Lit-14 | HighC Lit-5 | HighC Lit-5 |
| **5** | *MediumC Ortho-12* | LowC Lit-14 | HighC Lit-9 |
| **6** | HighC Lit-11 | **HighC Int-4** | **HighC Int-3** |
| **7** | HighC Lit-5 | *HighC Ortho-8* | **HighC Int-4** |
| **8** | **HighC Int-7** | HighC Lit-9 | **LowC Int-13** |
| **9** | *HighC Ortho-8* | HighC Lit-11 | HighC Lit-11 |
| **10** | HighC Lit-9 | *HighC Ortho-12* | LowC Lit-14 |
| **11** | *HighC Ortho-7* | LowC Lit-4 | *MediumC Ortho-18* |
| **12** | LowC Lit-4 | *LowC Ortho-7* | LowC Lit-4 |
| **13** | **HighC Int-3** | *MediumC Ortho-18* | *LowC Ortho-7* |
| **14** | *MediumC Ortho-18* | **HighC Int-3** | **MediumC Int-18** |
| **15** | **HighC Int-2** | **HighC Int-2** | **HighC Int-2** |
| **16** | HighC Lit-2 | HighC Lit-2 | HighC Lit-2 |
| ***MCODE*** |  |  |  |
| **Rank** | **cc**  **(cellular component)** | **bp**  **(biological process)** | **mf**  **(molecular function)** |
| **1** | **HighC Int-5** | **HighC Int-5** | *HighC Ortho-5* |
| **2** | *HighC Ortho-4* | *HighC Ortho-4* | LowC Lit-13 |
| **3** | *LowC Lit-13* | LowC Lit-13 | *HighC Ortho-4* |
| **4** | *HighC Ortho-5* | HighC Lit-4 | *LowC Ortho-10* |
| **5** | LowC Lit-11 | *HighC Ortho-5* | **HighC Int-5** |
| **6** | HighC Lit-4 | *LowC Ortho-10* | HighC Lit-4 |
| **7** | *LowC Ortho-2* | LowC Lit-11 | LowC Lit-11 |
| **8** | *LowC Ortho-10* | *LowC Ortho-2* | *LowC Ortho-2* |

*: when the number in parenthesis refers not to the size of the predicted module but to the number of proteins used by COFECO to annotate.

°: when the annotation is taken from GO Term Finder as COFECO is not able to annotate this modules.

HighC: High Confidence; MediumC: Medium Confidence; LowC: Low Confidence. Int is for **Int-P**I, Lit is for **Lit-PI**, Ortho is for **Ortho-PI** modules.
