# Supplementary material for: Inferring modules from human protein interactome classes
Source: BMC Syst Biol. 2010 Jul 23;4:102. doi: 10.1186/1752-0509-4-102 (PMC2923113; doi:10.1186/1752-0509-4-102)
Supplement: Additional file 2 — tableGOp-valuecomplete. Complete list of GO-annotated modules. [file 1752-0509-4-102-S2.DOC]

**Semi-quantitative evaluation of GO-annotated modules**

| **p-value**  **sorted by increasing order** | **cc**  **(cellular component)**  **-**  **number of matched proteins (size of predicted modules)** | **p-value**  **sorted by increasing order** | **bp**  **(biological process)** | **p-value**  **sorted by increasing**  **order** | **mf**  **(molecular function)** |
| --- | --- | --- | --- | --- | --- |
| **CFinder** |  |  |  |  |  |
| **LowC Int-13**  (5.722E-42) | GO:0005634: nucleus  GO:0005669: transcription factor TFIID complex  13(13) | **MediumC Int-18**  (2.59e-41)° | GO:0000502  [proteasome complex](http://amigo.geneontology.org/cgi-bin/amigo/go.cgi?view=details&query=GO:0000502) | **HighC Int-7**  (2.93e-38)° | GO:0003899 [DNA-directed RNA polymerase activity](http://amigo.geneontology.org/cgi-bin/amigo/go.cgi?view=details&query=GO:0003899)  15(19) |
| **MediumC Int-18**  (2.59e-41)° | GO:0000502: [proteasome complex](http://amigo.geneontology.org/cgi-bin/amigo/go.cgi?view=details&query=GO:0000502)  17(20) | **HighC Int-7**  (2.05e-35)° | GO:0000428 [DNA-directed RNA polymerase complex](http://amigo.geneontology.org/cgi-bin/amigo/go.cgi?view=details&query=GO:0000428)  13(19) | **HighC Int-3**  (8.475E-9) | GO:0003676: nucleic acid binding  GO:0003677: DNA binding  5(5) |
| **HighC Int-4**  (2.058E-36) | GO:0000139: Golgi membrane  GO:0005737: cytoplasm GO:0005794: Golgi apparatus  GO:0016020: membrane  GO:0030126: COPI vesicle coat  GO:0031410: cytoplasmic vesicle  9(9) | **LowC Int-13**  (1.691E-30) | GO:0006350: transcription  GO:0006355: regulation of transcription, DNA-dependent  GO:0006367: transcription initiation from RNA polymerase II promoter  11(13) | **LowC Int-13**  (1.536E-6) | [GO:0005515](javascript:go2GO("GO:0005515")): protein binding  12(13) |
| **HighC Int-7**  (2.05e-35)° | GO:0000428:[DNA-directed RNA polymerase complex](http://amigo.geneontology.org/cgi-bin/amigo/go.cgi?view=details&query=GO:0000428)  13(19) | **HighC Int-4**  (4.245E-23) | GO:0006810: transport  GO:0015031: protein transport  GO:0016192: vesicle-mediated transport  9(9) | **HighC Int-4**  (7.358E-6) | [GO:0005515](javascript:go2GO("GO:0005515")): protein binding  9(9) |
| **HighC Int-3**  (2.791E-11) | GO:0000775: chromosome, centromeric region  8407 GO:0005634: nucleus  4(5) | **HighC Int-3**  (4.397E-8) | [GO:0045449](javascript:go2GO("GO:0045449")):  regulation of transcription  5(5) | **HighC Int-2**  (0.030) | [GO:0005515](javascript:go2GO("GO:0005515")): protein binding  4(5) |
| **HighC Int-2**  (5.105E-4) | [GO:0005634](javascript:go2GO("GO:0005634")): nucleus  5(5) | **HighC Int-2**  (1.030E-4) | [GO:0006355](javascript:go2GO("GO:0006355")): regulation of transcription, DNA-dependent  4(5) | **MediumC Int-18**  (0.00026)° | GO:0016887 [ATPase activity](http://amigo.geneontology.org/cgi-bin/amigo/go.cgi?view=details&query=GO:0016887)  5(20) |
| **LowC Lit-14**  (1.995E-35) | GO:0005634: nucleus  GO:0005669: transcription factor TFIID complex  11(11) | **HighC Lit-5**  (3.24e-25)° | GO:0005665:[DNA-directed RNA polymerase II, core complex](http://amigo.geneontology.org/cgi-bin/amigo/go.cgi?view=details&query=GO:0005665)  8(9) | **HighC Lit-5**  (8.20e-21)° | GO:0003899  [DNA-directed RNA polymerase activity](http://amigo.geneontology.org/cgi-bin/amigo/go.cgi?view=details&query=GO:0003899)  8(9) |
| **HighC Lit-11**  (8.935E-28) | GO:0000178: exosome (RNase complex)  GO:0005634: nucleus  8(11) | **HighC Lit-14**  (1.045E-24) | GO:0006350: transcription  GO:0006355: regulation of transcription, DNA-dependent  GO:0006367: transcription initiation from RNA polymerase II promoter  9(11) | **HighC Lit-9**  (3.077E-17) | GO:0003723: RNA binding  GO:0005515: protein binding  8(8) |
| **HighC Lit-9**  (1.411E-16) | GO:0005634: nucleus  GO:0030529: ribonucleoprotein complex  7(8) | **HighC Lit-9**  (6.651E-19) | GO:0006397: mRNA processing  GO:0008380: RNA splicing  8(8) | **HighC Lit-11**  (1.644E-5) | [GO:0005515](javascript:go2GO("GO:0005515")): protein binding  10(11)* |
| **HighC Lit-5**  (3.24e-25)° | GO:0005665[DNA-directed RNA polymerase II, core complex](http://amigo.geneontology.org/cgi-bin/amigo/go.cgi?view=details&query=GO:0005665)  8(9) | **HighC Lit-11**  (9.636E-19) | [GO:0006364](javascript:go2GO("GO:0006364")): rRNA processing  8(11)° | **LowC Lit-14**  (1.849E-5) | [GO:0005515](javascript:go2GO("GO:0005515")): protein binding  10(11) |
| **LowC Lit-4**  (6.864E-13) | GO:0005634: nucleus  GO:0035267: NuA4 histone acetyltransferase complex  4(15) | **LowC Lit-4**  (1.113E-17) | GO:0006350: transcription  GO:0006355: regulation of transcription, DNA-dependent  14(14)* | **LowC Lit-4**  (9.489E-5) | [GO:0005515](javascript:go2GO("GO:0005515")): protein binding  11(14)* |
| **HighC Lit-2**  (5.105E-4) | [GO:0005634](javascript:go2GO("GO:0005634")): nucleus  5(5) | **HighC Lit-2**  (1.030E-4) | [GO:0006355](javascript:go2GO("GO:0006355")): regulation of transcription, DNA-dependent  4(5) | **HighC Lit-2**  (0.030) | [GO:0005515](javascript:go2GO("GO:0005515")): protein binding  4(5) |
| **HighC Ortho-8**  (8.90e-33)° | GO:0000428  [DNA-directed RNA polymerase complex](http://amigo.geneontology.org/cgi-bin/amigo/go.cgi?view=details&query=GO:0000428)  12(17) | **HighC Ortho-8**  (3.71e-20)° | GO:0006350  [transcription](http://amigo.geneontology.org/cgi-bin/amigo/go.cgi?view=details&query=GO:0006350)  13(17) | **HighC Ortho-8**  (4.12e-36)° | GO:0003899 [DNA-directed RNA polymerase activity](http://amigo.geneontology.org/cgi-bin/amigo/go.cgi?view=details&query=GO:0003899)  14(17) |
| **HighC Ortho-12**  (1.334E-30) | GO:0005634: nucleus  GO:0005737: cytoplasm GO:0005829: cytosol  GO:0005839: proteasome core complex  GO:0043234: protein complex  10(10) | **HighC Ortho-12**  (8.465E-18) | [GO:0006511](javascript:go2GO("GO:0006511")): ubiquitin-dependent protein catabolic process  10(10) | **HighC Ortho-12**  (2.600E-31) | GO:0004298: threonine-type endopeptidase activity  GO:0008233: peptidase activity  GO:0016787: hydrolase activity  10(10) |
| **LowC Ortho-7**  (4.106E-13) | GO:0000178: exosome (RNase complex)  GO:0005634: nucleus  GO:0005737: cytoplasm  4(8) | **LowC Ortho-7**  (1.976E-14) | [GO:0006364](javascript:go2GO("GO:0006364")): rRNA processing  6(8) | **MediumC Ortho-18**  (2.770E-5) | [GO:0005515](javascript:go2GO("GO:0005515")): protein binding  12(15)* |
| **MediumC Ortho-18** (6.025E-10) | [GO:0005634](javascript:go2GO("GO:0005634")): nucleus  14(14) | **MediumC Ortho-18** (4.910E-12) | [GO:0042254](javascript:go2GO("GO:0042254")): ribosome biogenesis  5(11)* | **LowC Ortho-7**  (8.303E-4) | [GO:0005515](javascript:go2GO("GO:0005515")): protein binding  7(8) |
| **MCODE** |  |  |  |  |  |
| **HighC Int-5**  (2.058E-36) | GO:0000139: Golgi membrane  GO:0005737: cytoplasm  GO:0005794: Golgi apparatus GO:0016020: membrane  GO:0030126: COPI vesicle coat  GO:0031410: cytoplasmic vesicle  9(9) | **HighC Int-5**  (1.984E-29) | GO:0006810: transport  GO:0006890: retrograde vesicle-mediated transport, Golgi to ER  GO:0015031: protein transport  GO:0016192: vesicle-mediated transport  8(9) | **HighC Int-5**  (7.358E-6) | [GO:0005515](javascript:go2GO("GO:0005515")): protein binding  9(9) |
| **LowC Lit-13**  (3.687E-29) | GO:0005634: nucleus  GO:0005669: transcription factor TFIID complex  11(23)* | **LowC Lit-13**  (7.666E-20) | GO:0006350: transcription GO:0006355: regulation of transcription, DNA-dependent  19(23)* | **LowC Lit-13**  (1.023E-8) | [GO:0005515](javascript:go2GO("GO:0005515")): protein binding  19(22) |
| **LowC Lit-11**  (5.171E-18) | GO:0000119: mediator complex  GO:0005634: nucleus  6(9) | **HighC Lit-4**  (1.567E-17) | [GO:0008033](javascript:go2GO("GO:0008033")): tRNA processing  7(9)* | **HighC Lit-4**  (2.736E-5) | [GO:0005515](javascript:go2GO("GO:0005515")): protein binding  8(8) |
| **HighC Lit-4**  (9.858E-14) | GO:0005634: nucleus  GO:0005655: nucleolar ribonuclease P complex  4(9) | **LowC Lit-11**  (1.763E-9) | GO:0006350: transcription GO:0006355: regulation of transcription, DNA-dependent  8(9) | **LowC Lit-11**  (2.736E-5) | [GO:0005515](javascript:go2GO("GO:0005515")): protein binding  8(8) |
| **HighC Ortho-4**  (1.559E-29) | GO:0000139: Golgi membrane  GO:0005737: cytoplasm GO:0005794: Golgi apparatus  GO:0016020: membrane  GO:0030126: COPI vesicle coat  GO:0031410: cytoplasmic vesicle  8(11)* | **HighC Ortho-4**  (7.483E-27) | GO:0006810: transport  GO:0006886: intracellular protein transport  GO:0006890: retrograde vesicle-mediated transport, Golgi to ER  GO:0015031: protein transport  GO:0016192: vesicle-mediated transport  7(10)* | **HighC Ortho-5**  (2.144E-15) | [GO:0003735](javascript:go2GO("GO:0003735")): structural constituent of ribosome  8(9) |
| **HighC Ortho-5**  (3.688E-21) | GO:0005622: intracellular  GO:0005829: cytosol  GO:0005840: ribosome GO:0030529: ribonucleoprotein complex  8(9) | **HighC Ortho-5**  (1.831E-16) | [GO:0006412](javascript:go2GO("GO:0006412")): translation  9(9) | **LowC Ortho-10**  (3.42e-07)° | GO:0003723 [RNA binding](http://amigo.geneontology.org/cgi-bin/amigo/go.cgi?view=details&query=GO:0003723)  7(11) |
| **LowC Ortho-2**  (2.678E-12) | GO:0005634: nucleus GO:0005669: transcription factor TFIID complex  4(5)* | **LowC Ortho-10**  (6.35e-12)° | GO:0006397 [mRNA processing](http://amigo.geneontology.org/cgi-bin/amigo/go.cgi?view=details&query=GO:0006397)  8(11) | **HighC Ortho-4**  (5.317E-7) | [GO:0005515](javascript:go2GO("GO:0005515")): protein binding  11(11)* |
| **LowC Ortho-10**  (2.01e-06)° | GO:0005634 [nucleus](http://amigo.geneontology.org/cgi-bin/amigo/go.cgi?view=details&query=GO:0005634)  11(11) | **LowC Ortho-2**  (6.867E-5) | GO:0006350: transcription GO:0006355: regulation of transcription, DNA-dependent  4(5)* | **LowC Ortho-2**  (0.041) | [GO:0005515](javascript:go2GO("GO:0005515")): protein binding  4(5)* |

*: when the number in parenthesis refers not to the size of the predicted module but to the number of proteins used by COFECO to annotate.

°: when the annotation is taken from GO Term Finder as COFECO is not able to annotate the module.

**HighC**: High Confidence

**MediumC**: Medium Confidence

**LowC**: Low Confidence

***Go Term Finder* p-value.**

***p*_*value* = ∑**

**n**

**j = x**

____________

( ) ( )

( )

M

j

N

n

N-M

n-j

It calculates the p-value as the probability of seeing ***x*** (or more) ***M***-annotated genes, out of ***n*** genes in a module,

while considering a population of ***N***-annotated genes.

***N*** is the number of annotated genes from the *gene_association.goa_human* file (*)

***M*** is the number of genes with a particular annotation from the *gene_association.goa_human* file (example: GO:0000139: Golgi membrane )

***n*** is the number of genes in the module

***x*** is the number of genes with ***M*** annotation in the module of ***n*** genes

* UniProtKB-GOA (GO Annotation@EBI) (<http://www.ebi.ac.uk/GOA/>).

To annotate our set of genes we used the default statistics (Hypergeometric statistical test, Bonferroni correction and FDR correction, p-value cutoff = 0.01)

Extended presentation of p-values is reported in *annotation_description.doc* file.

***COFECO*** computed p-values also used the default statistics (Hypergeometric statistical test***,*** FDR correction***,*** p-value cutoff = 1).

Extended presentation of *COFECO* p-values is also reported in *annotation_description.doc* file, together with Bonferroni and FDR corrections.
