# Supplementary material for: Inferring modules from human protein interactome classes
Source: BMC Syst Biol. 2010 Jul 23;4:102. doi: 10.1186/1752-0509-4-102 (PMC2923113; doi:10.1186/1752-0509-4-102)
Supplement: Additional file 3 — overlapscores. Overlap scores computed for MCODE and CFinder modules. [file 1752-0509-4-102-S3.DOC]

***CFinder* summary**

| **Confidence**  **Level** | **Module** | **a = predicted module size** | **i =**  **intersection**  **between module & complex** | **b = known complex**  **size** | **Annotated MIPS**  **or Reactome**  **Complexes** | **Overlap Score**  **ω= i^2^/w**  i = a ∩ b  w = a * b | **Jaccard index**  **J = i/ u**  i = a ∩ b  u = a U b | **Sorensen index**  **S = 2i/c**  i = a ∩ b  c = a+b |
| --- | --- | --- | --- | --- | --- | --- | --- | --- |
| High | Int-2 | 5 | 5 | 10 | H3.1 complex | 0.50 | 0.50 | 0.67 |
|  |  | 5 | 5 | 6 | ASF1-interacting protein complex | 0.83 | 0.83 | 0.90 |
|  | Int-3 | na |  |  |  |  |  |  |
|  | Int-4 | 7° | 7 | 8 | Coatomer:Arf1-GTP Complex [cytosol] (**Reactome**) | 0.88 | 0.88 | 0.94 |
|  | Int-7 | 19 | 5 | 25 | TNF-alpha/NF-kappa B signaling complex 5 | 0.05 | 0.13 | 0.23 |
|  |  |  | 5 | 12 | RNA polymerase II core complex | 0.11 | 0.19 | 0.32 |
| Medium | Int-18 | 20 | 9 | 22 | 26S proteasome | 0.18 | 0.27 | 0.42 |
|  |  |  | 2 | 16 | PA28-20S proteasome | 0.01 | 0.06 | 0.11 |
|  |  |  | 16 | 36 | PA700-20S-PA28 complex | 0.36 | 0.4 | 0.57 |
|  |  |  | 2 | 15 | PA28gamma-20S proteasome | 0.01 | 0.06 | 0.11 |
| Low | Int-13 | 13 | 12 | 12 | TFIID-beta complex | 0.92 | 0.92 | 0.96 |
|  |  |  |  | 15 | DA complex | 0.74 | 0.75 | 0.86 |
| High | Lit-2 | 5 | 5 | 10 | H3.1 complex | 0.50 | 0.50 | 0.67 |
|  |  | 5 | 5 | 6 | ASF1-interacting protein complex | 0.83 | 0.83 | 0.90 |
|  | Lit-5 | na |  |  |  |  |  |  |
|  | Lit-9 | 8 | 7 | 7 | LSm1-7 complex | 0.88 | 0.88 | 0.94 |
|  | Lit-11 | 10 | 8 | 11 | Exosome | 0.58 | 0.62 | 0.76 |
| Low | Lit-4 | 15* | 10 | 15 | NuA4/Tip60-HAT complex A | 1 | 1 | 1 |
|  | Lit-14 | 11 | 10 | 12 | TFIID-beta complex | 0.76 | 0.77 | 0.87 |
|  |  |  |  | 15 | DA complex | 0.61 | 0.61 | 0.77 |
|  |  |  |  | 16 | DAB complex | 0.57 | 0.59 | 0.74 |
| High | Ortho-8 | 17 | 3 | 25 | TNF-alpha/NF-kappa B signaling complex 5 | 0.02 | 0.08 | 0.14 |
|  |  |  | 4 | 12 | RNA polymerase II core complex | 0.07 | 0.16 | 0.28 |
|  | Ortho-12 | 9* | 9 | 22 | 26S proteasome | 0.41 | 0.41 | 0.58 |
|  |  |  |  | 14 | 20S proteasome | 0.64 | 0.64 | 0.78 |
|  |  |  |  | 16 | PA28-20S proteasome | 0.56 | 0.56 | 0.72 |
|  |  |  |  | 36 | PA700-20S-PA28 complex | 0.25  0.23 | 0.25  0.24 | 0.40  0.39 |
|  |  |  |  | 15 | PA28gamma-20S proteasome | 0.6 | 0.6 | 0.75 |
| Medium | Ortho-18 | 19 | 9 | 22 | 26S proteasome | 0.19 | 0.28 | 0.43 |
|  |  |  | 2 | 16 | PA28-20S proteasome | 0.01 | 0.06 | 0.11 |
|  |  |  | 16 | 36 | PA700-20S-PA28 complex | 0.37 | 0.41 | 0.58 |
|  |  |  | 2 | 15 | PA28gamma-20S proteasome | 0.01 | 0.06 | 0.12 |
| Low | Ortho-7 | 7* | 4 | 11 | Exosome | 0.21 | 0.36 | 0.45 |

***MCODE* summary**

| **Confidence**  **Level** | **Module** | **a = predicted module size** | **i =**  **intersection**  **between module & complex** | **b = known complex**  **size** | **Annotated MIPS or Reactome**  **Complexes** | **Overlap Score**  **ω= i^2^/w**  i = a ∩ b  w = a * b | **Jaccard index**  **J = i/ u**  i = a ∩ b  u = a U b | **Sorensen index**  **S = 2i/c**  i = a ∩ b  c = a+b |
| --- | --- | --- | --- | --- | --- | --- | --- | --- |
| High | Int-5 | 7° | 7 | 8 | Coatomer:Arf1-GTP Complex [cytosol] (**Reactome**) | 0.88 | 0.88 | 0.94 |
| High | Lit-4 | 7 | 7 | 10 | Rnase/Mrp complex | 0.70 | 0.70 | 0.82 |
| Low | Lit-11 | 9 | 9 | 32 | Mediator complex | 0.28 | 0.28 | 0.44 |
|  | Lit-13 | 22 | 10 | 12 | TFIID-beta complex | 0.38 | 0.42 | 0.59 |
|  |  |  |  | 16 | DAB complex | 0.28 | 0.36 | 0.53 |
| High | Ortho-4 | 9° | 6 | 8 | Coatomer:Arf1-GTP Complex [cytosol] (**Reactome**) | 0.50 | 0.55 | 0.71 |
|  | Ortho-5 | 9° | 8 | 82 | 80S Ribosome:mRNA:peptidyl-tRNA with elongating peptide [cytosol] (**Reactome**) | 0.09 | 0.09 | 0.18 |
| Low | Ortho-2 | 4* | 4 | 12 | TFIID-beta complex | 0.34 | 0.34 | 0.50 |
|  | Ortho-10 | 11 | 5 | 5 | Cleavage and polyadenylation factor (CPSF) | 0.45 | 0.45 | 0.63 |

| ***:** when **a** refers not to the original predicted module but to the number of proteins used by COFECO to annotate |
| --- |
| **°:** from Reactome db when CORUM annotation is absent |
