# Supplementary material for: Inferring modules from human protein interactome classes
Source: BMC Syst Biol. 2010 Jul 23;4:102. doi: 10.1186/1752-0509-4-102 (PMC2923113; doi:10.1186/1752-0509-4-102)
Supplement: Additional file 4 — tablePathways. Pathway annotation and evaluation for MCODE and CFinder modules. [file 1752-0509-4-102-S4.DOC]

| ***CFinder*** |  |  |  |  |  |  |  |
| --- | --- | --- | --- | --- | --- | --- | --- |
| **Confidence**  **Level** | **Module Name** | **predicted module**  **size** | **Pathways** | **number of matched proteins (size of predicted module)** | **p-value**  **(FDR-corrected)** | **Cohesiveness** | **Cross-talk** |
| High | Int-2 | 5 | Control  (PathwaysInteractionDatabase) | 2(5) | - | Sparse | High |
|  | Int-3 | 5 | na |  |  | - | - |
|  | Int-4 | 9 | COPI Mediated Transport  (Reactome) | 6(9) | - | Dense | No |
|  | Int-7 | 17 | RNA polymerase  (kegg)  RNA polymerase I transcription initiation  (Reactome) | 4(17)  4(21) |  | Dense | High |
| Medium | Int-18 | 18 | Proteasome  (kegg)  Regulation of activated PAK-2p34 by proteasome mediated degradation | 16(18)  12(18) | - | Dense | No |
| Low | Int-13 | 13 | Basal transcription factors  (COFECO- kegg) | 9(10) | 2.028E-16 | Dense | High |
| High | Lit-2 | 5 | Control  (PathwaysInteractionDatabase) | 2(5) | - | - | - |
|  | Lit-5 | 9 | RNA polymerase  (kegg) | 8(9) | - | Dense | High |
|  | Lit-9 | 8 | RNA degradation  Spliceosome  (kegg) | 7(8)  6(8) | - | - | - |
|  | Lit-11 | 10 | RNA degradation  (kegg) | 8(10) | - | - | - |
| Low | Lit-4 | 16 | Wnt signaling pathway  (kegg) | 2(16) | - | Sparse | No |
|  | Lit-14 | 11 | Basal transcription factors  (COFECO:kegg) | 7(8) | 8.827E-13 | Dense | High |
| High | Ortho-8 | 15 | RNA polymerase  (kegg) | 15(15) | - | Dense | High |
|  | Ortho-12 | 10 | Proteasome  (COFECO-kegg) | 9(10) | 1.030E-15 | Dense | No |
| Medium | Ortho-18 | 17 | Proteasome  (kegg) | 16(18) | - | Dense | No |
| Low | Ortho-7 | 8 | RNA degradation  (kegg) | 4(8) | - | - | - |
| ***MCODE*** |  |  |  |  |  |  |  |
| **Confidence**  **Level** | **Module Name** | **predicted module**  **size** | **Pathways** | **number of matched proteins (predicted module)** | **p-value**  **(FDR-corrected)** | **Cohesiveness** | **Cross-talk** |
| High | Int-5 | 9 | Control  (PathwaysInteractionDatabase) | 5(9) | - | - | - |
| High | Lit-4 | 7 | na |  | - | - | - |
| Low | Lit-11 | 9 | na |  | - | Dense | High |
|  | Lit-13 | 22 | Basal transcription factors  (COFECO-kegg) | 7(13) | 1.783E-10 | Dense | High |
| High | Ortho-4 | 12 | RNA degradation  (kegg)  COPI Mediated Transport  (Reactome) | 3(12)  5(12) | -  - | Dense | No |
|  | Ortho-5 | 9 | Ribosome  (COFECO-kegg) | 4(4) | 3.553E-6 | Dense | No |
| Low | Ortho-2 | 6 | Basal transcription factors  (COFECO-kegg) | 4(5) | 2.069E-7 | Sparse | No |
|  | Ortho-10 | 11 | RNA degradation  (kegg) | 2(11) | - | Sparse | No |

**Pathways are taken from COFECO and from KEGG when COFECO is not able to annotate the module.**

**When pathways are taken from KEGG, a p-value is not computed.**

**Extended presentation of related p-values is reported in *annotation_description.doc* file.**
