# Supplementary material for: Inferring modules from human protein interactome classes
Source: BMC Syst Biol. 2010 Jul 23;4:102. doi: 10.1186/1752-0509-4-102 (PMC2923113; doi:10.1186/1752-0509-4-102)
Supplement: Additional file 5 — TablesModulewisePvsR. P vs R scores computed for MOCDE and CFinder modules. [file 1752-0509-4-102-S5.DOC]

**CFinder**

| **modules** | n° of proteins in  predicted module | n° of proteins matched in MIPS complex | n° of proteine of MIPS complex | **MIPS complex name** | frequency  for each  MIPS complex | mean values | **P** | **R** |
| --- | --- | --- | --- | --- | --- | --- | --- | --- |
| **Lit** |  |  |  |  |  |  | **0,83** | **0,86** |
| **Lit-2 (High)**  **GO:**  chromatin remodeling complex  **p-value:** 0.000001 |  |  |  |  |  |  |  |  |
| Lit-2 | 5 | 2 | 3 | Chromatin assembly complex | 0,67 | 0,68 |  |  |
| Lit-2 | 5 | 5 | 10 | Histone H3.1 complex | 0,5 | 0,68 |  |  |
| Lit-2 | 5 | 5 | 7 | ASF-1 histone containing complex | 0,71 | 0,68 |  |  |
| Lit-2 | 5 | 5 | 6 | ASF-1 interacting protein complex | 0,84 | 0,68 |  |  |
| **Lit-9 (High)**  **GO:**  ribonucleoprotein complex  **p-value:**  0 |  |  |  |  |  |  |  |  |
| Lit-9 | 8 | 7 | 7 | LSM 1-7 complex | 1 | 1 |  |  |
| Lit-9 | 8 | 7 | 7 | LSM 2-8 complex | 1 | 1 |  |  |
| **Lit-5 (High)**  **GO:**  RNA polymerase complex  **p-value:**  **0** |  |  |  |  |  |  |  |  |
| Lit-5 | 9 | 8 | 24 | RNA polymerase II holoenzyme complex | 0,34 | 0,49 |  |  |
| Lit-5 | 9 | 8 | 12 | RNA polymerase II core complex | 0,67 | 0,49 |  |  |
| Lit-5 | 9 | 8 | 17 | RNAPII | 0,47 | 0,49 |  |  |
| **Lit-11 (High)**  **GO:**  exosome  **p-value:**  1.61E-022 |  |  |  |  |  |  |  |  |
| Lit-11 | 10 | 7 | 10 | Exosome | 0,7 | 0,7 |  |  |
| **Lit-14 (Low)**  **GO:**  transcription factor  TFIID complex  **p-value:**  1.38e-29 |  |  |  |  |  |  |  |  |
| Lit-14 | 11 | 9 | 10 | TFIID complex | 0,9 | 0,95 |  |  |
| Lit-14 | 11 | 5 | 5 | TFIID subcomplex | 1 | 0,95 |  |  |
| **Lit-4 (Low)**  **GO:**  H4/H2A histone  acetyltransferase complex  **p-value:**  5.80e-15 |  |  |  |  |  |  |  |  |
| Lit-4 | 16 | 4 | 5 | TIP60 HAT complex | 0,8 | 0,7 |  |  |
| Lit-4 | 16 | 6 | 10 | NuA4/TIP 60 HAT complex | 0,6 | 0,7 |  |  |
| **Ortho** |  |  |  |  |  |  | **0,25** | **0,25** |
| **Ortho-8 (High)**  **GO:**  RNA polymerase complex  **p-value:**  1.42E-025 |  |  |  |  |  |  |  |  |
| Ortho-8 | 17 | 3 | 24 | RNA polymerase II holoenzyme complex | 0,13 | 0,24 |  |  |
| Ortho-8 | 17 | 4 | 12 | RNA polymerase II core complex | 0,34 | 0,24 |  |  |
| Ortho-8 | 17 | 4 | 17 | RNAPII | 0,24 | 0,24 |  |  |
| **Ortho-12 (High)**  **GO:**  proteasome core complex  **p-value:**  2.89E-027 |  |  |  |  |  |  |  |  |
| Ortho-12 | 11 | 8 | 14 | 20S proteasome | 0,57 | 0,47 |  |  |
| Ortho-12 | 11 | 9 | 22 | 26S proteasome | 0,41 | 0,47 |  |  |
| Ortho-12 | 11 | 9 | 36 | PA700-20S-PA28 complex | 0,25 | 0,47 |  |  |
| Ortho-12 | 11 | 8 | 16 | PA28-20S proteasome | 0,5 | 0,47 |  |  |
| Ortho-12 | 11 | 9 | 15 | PA28 gamma-20S proteasome | 0,6 | 0,47 |  |  |
| Ortho-12 | 11 | 3 | 6 | Ubiquitin proteasome complex | 0,5 | 0,47 |  |  |
| **Ortho-18 (Medium)**  **GO:**  proteasome complex  **p-value:**  1.60e-42 |  |  |  |  |  |  |  |  |
| Ortho-18 | 19 | 13 | 20 | PA700 complex | 0,65 | 0,33 |  |  |
| Ortho-18 | 19 | 13 | 36 | PA700-20S PA28 complex | 0,36 | 0,33 |  |  |
| Ortho-18 | 19 | 8 | 22 | 26S proteasome | 0,36 | 0,33 |  |  |
| Ortho-18 | 19 | 2 | 14 | 20S proteasome | 0,14 | 0,33 |  |  |
| Ortho-18 | 19 | 2 | 16 | PA28-20S proteasome | 0,13 | 0,33 |  |  |
| Ortho-18 | 19 | 2 | 15 | PA28 gamma -20S proteasome | 0,13 | 0,33 |  |  |
| Ortho-18 | 19 | 1 | 6 | Ubiquitin proteasome complex | 0,17 | 0,33 |  |  |
| **Ortho-7 (Low)**  **GO:**  exosome  **p-value:**  1.31e-11 |  |  |  |  |  |  |  |  |
| Ortho-7 | 8 | 4 | 10 | Exosome | 0,4 | 0,6 |  |  |
| Ortho-7 | 8 | 1 | 10 | RNase/Mrp complex | 0,1 | 0,6 |  |  |
| Ortho-7 | 8 | 4 | 10 | Exosome | 0,4 | 0,6 |  |  |
| Ortho-7 | 8 | 1 | 10 | mRNA decay complex | 0,1 | 0,6 |  |  |
| **Int** |  |  |  |  |  |  | **0,4** | **0,69** |
| **Int-2 (High)**  **GO:**  chromatin remodeling complex  **p-value**  0.000001 |  |  |  |  |  |  |  |  |
| Int-2 | 5 | 2 | 3 | Chromatin assembly complex | 0,67 | 0,64 |  |  |
| Int-2 | 5 | 5 | 10 | Histone H3.1 complex | 0,5 | 0,64 |  |  |
| Int-2 | 5 | 4 | 7 | ASF-1 histone containing complex | 0,57 | 0,64 |  |  |
| Int-2 | 5 | 5 | 6 | ASF-1 interacting protein complex | 0,83 | 0,64 |  |  |
| **Int-3 (High)**  **GO:**  chromosome centromeric region  **p-value:**  0 |  |  |  |  |  |  |  |  |
| Int-3 | 5 | 1 | 37 | CEN complex | 0,03 | 0,03 |  |  |
| **Int-7 (High)**  **GO:**  RNA polymerase complex  **p-value:**  6.95E-025 |  |  |  |  |  |  |  |  |
| Int-7 | 19 | 5 | 24 | RNA polymerase II holoenzyme complex | 0,21 | 0,31 |  |  |
| Int-7 | 19 | 5 | 12 | RNA polymerase II core complex | 0,42 | 0,31 |  |  |
| Int-7 | 19 | 5 | 17 | RNAPII | 0,29 | 0,31 |  |  |
| **Int-18 (Medium)**  **GO:**  proteasome complex  **p-value:**  1.06e-41 |  |  |  |  |  |  |  |  |
| Int-18 | 20 | 2 | 14 | 20S proteasome | 0,5 | 0,33 |  |  |
| Int-18 | 20 | 9 | 22 | 26S proteasome | 0,41 | 0,33 |  |  |
| Int-18 | 20 | 13 | 36 | PA700-20S-PA28 complex | 0,36 | 0,33 |  |  |
| Int-18 | 20 | 12 | 20 | PA700 complex | 0,6 | 0,33 |  |  |
| Int-18 | 20 | 2 | 16 | PA28-20S proteasome | 0,13 | 0,33 |  |  |
| Int-18 | 20 | 2 | 15 | PA28 gamma -20S proteasome | 0,13 | 0,33 |  |  |
| Int-18 | 20 | 1 | 6 | Ubiquitin proteasome complex | 0,17 | 0,33 |  |  |
| **Int-13 (Low)**  **GO:**  transcription factor  TFIID complex  **p-value:**  5.58e-36 |  |  |  |  |  |  |  |  |
| Int-13 | 13 | 10 | 11 | TFIID complex | 0,91 | 0,97 |  |  |
| Int-13 | 13 | 5 | 5 | TFIID subcomplex | 1 | 0,97 |  |  |
| Int-13 | 13 | 5 | 5 | Transcription initiation factor complex | 1 | 0,97 |  |  |

**MCODE**

| **modules** | n° of proteins in  predicted module | n° of proteins matched in MIPS complex | n° of proteine of MIPS complex | **MIPS complex name** | frequency  for each  MIPS complex | mean values | **P** | **R** |
| --- | --- | --- | --- | --- | --- | --- | --- | --- |
| **Lit** |  |  |  |  |  |  | **0,67** | **0,25** |
| **Lit-4 (High)**  **GO:**  nucleolar ribonuclease P-complex  **p-value**  6.91E-017 |  |  |  |  |  |  |  |  |
| Lit-4 | 7 | 6 | 10 | RNase/Mrp complex | 0,6 | 0,6 |  |  |
| **Lit-11 (Low)**  **GO:**  S mediator complex  **p-value**  1.18e-13 |  |  |  |  |  |  |  |  |
| Lit-11 | 9 | 1 | 3 | MED18-MED20-MED29 mediator subcomplex | 0,33 | 0,16 |  |  |
| Lit-11 | 9 | 3 | 8 | CRSP mediator complex | 0,34 | 0,16 |  |  |
| Lit-11 | 9 | 2 | 13 | ARC92 mediator complex | 0,15 | 0,16 |  |  |
| Lit-11 | 9 | 1 | 5 | Mediator complex 1 | 0,2 | 0,16 |  |  |
| Lit-11 | 9 | 1 | 3 | Mediator complex 2 | 0,33 | 0,16 |  |  |
| Lit-11 | 9 | 3 | 15 | ARC complex | 0,2 | 0,16 |  |  |
| Lit-11 | 9 | 2 | 14 | DRIP complex | 0,14 | 0,16 |  |  |
| Lit-11 | 9 | 4 | 16 | TRAP complex | 0,25 | 0,16 |  |  |
| Lit-11 | 9 | 5 | 14 | SMCC complex | 0,36 | 0,16 |  |  |
| Lit-11 | 9 | 3 | 12 | Pc2 complex | 0,25 | 0,16 |  |  |
| Lit-11 | 9 | 4 | 10 | TRAPP-SMCC mediator complex | 0,4 | 0,16 |  |  |
| Lit-11 | 9 | 4 | 32 | Mediator complex | 0,13 | 0,16 |  |  |
| **Lit-13 (Low)**  **GO:**  transcription factor  TFIID complex  **p-value:**  4.81e-25 |  |  |  |  |  |  |  |  |
| Lit-13 | 21 | 9 | 10 | TFIID complex | 0,9 | 0,83 |  |  |
| Lit-13 | 21 | 5 | 5 | TFIID subcomplex | 1 | 0,83 |  |  |
| Lit-13 | 21 | 3 | 5 | Transcription initiation factor complex | 0,6 | 0,83 |  |  |
| **Ortho** |  |  |  |  |  |  | **0,5** | **0,23** |
| **Ortho-4 (High)**  **GO:**  COPI vesicle coat  **p-value**  3.49E-020 |  |  |  |  |  |  |  |  |
| Ortho-4 |  |  |  | na |  |  |  |  |
| **Ortho-5 (High)**  **GO:**  large ribosomal subunit  **p-value:**  1.78e-11 |  |  |  |  |  |  |  |  |
| Ortho-5 | 9 | 1 | 34 | 40S ribosomal subunit, cytoplasmic | 0,33 | 0,19 |  |  |
| Ortho-5 | 9 | 7 | 80 | Ribosome | 0,09 | 0,19 |  |  |
| Ortho-5 | 9 | 7 | 47 | 60S ribosomal subunit, cytoplasmic | 0,15 | 0,19 |  |  |
| **Ortho-2 (Low)**  **GO:**  transcription factor  TFIID complex  **p-value:**  4.01e-10 |  |  |  |  |  |  |  |  |
| Ortho-2 | 7 | 3 | 10 | TFIID complex | 0,3 | 0,43 |  |  |
| Ortho-2 | 7 | 3 | 5 | TFIID subcomplex | 0,6 | 0,43 |  |  |
| Ortho-2 | 7 | 2 | 5 | Transcription initiation factor complex | 0,4 | 0,43 |  |  |
| **Ortho-10 (Low)**  **GO:**  Nucleus  **p-value:**  0.00206 |  |  |  |  |  |  |  |  |
| Ortho-10 | 11 | 5 | 5 | CPSF complex | 1 | 0,5 |  |  |
| Ortho-10 | 11 | 3 | 7 | Polyadenylation complex | 0,43 | 0,5 |  |  |
| Ortho-10 | 11 | 2 | 30 | CDC5L complex | 0,07 | 0,5 |  |  |
| **Int** |  |  |  |  |  |  | **0,34** | **0,13** |
| **Int-5 (High)**  **GO:**  COPI vesicle coat  **p-value:**  1.98E-025 |  |  |  |  |  |  |  |  |
| Int-5 |  |  |  | na |  |  |  |  |
| **Int-3 (--)**  **GO:**  Nucleolus  **p-value:**  4.88e-17 |  |  |  |  |  |  |  |  |
| Int-3 | 13 | 5 | 104 | Nap56p-associated pre-rRNA complex | 0,05 | 0,05 |  |  |
| **Int-10 (--)**  **GO:**  [Arp2/3 protein complex](http://amigo.geneontology.org/cgi-bin/amigo/go.cgi?view=details&query=GO:0005885)  **p-value:**  1.18e-19 |  |  |  |  |  |  |  |  |
| Int-10 | 10 | 6 | 7 | Arp2/3 protein complex | 0,86 | 0,86 |  |  |
| **Int-14 (--)**  **GO:**  Ribosome  **p-value:**  8.92e-17 |  |  |  |  |  |  |  |  |
| Int-14 | 31 | 7 | 48 | 39S ribosomal subunit, mitochondrial | 0,15 | 0,1 |  |  |
| Int-14 | 31 | 5 | 80 | Ribosome, cytoplasmic | 0,06 | 0,1 |  |  |
| Int-14 | 31 | 4 | 47 | 60S ribosomal subunit, cytoplasmic | 0,09 | 0,1 |  |  |
| Int-14 | 31 | 1 | 34 | 40S ribosomal subunit, cytoplasmic | 0,03 | 0,1 |  |  |
| Int-14 | 31 | 7 | 78 | 55S ribosome, mitochondrial | 0,09 | 0,1 |  |  |
| Int-14 | 31 | 7 | 48 | 39S ribosomal subunit, mitochondrial | 0,15 | 0,1 |  |  |
