# Supplementary material for: Inferring modules from human protein interactome classes
Source: BMC Syst Biol. 2010 Jul 23;4:102. doi: 10.1186/1752-0509-4-102 (PMC2923113; doi:10.1186/1752-0509-4-102)
Supplement: Additional file 6 — TablesProteinwisePvsR. P vs R scores computed for MOCDE and CFinder modules (disaggregated by interactome class). [file 1752-0509-4-102-S6.DOC]

**Literature CFinder**

| **Modules** | n° of proteins in  predicted module | n° of proteins matched in MIPS complex | n° of protein of MIPS complex | **MIPS complex name** | **P** | **R** |
| --- | --- | --- | --- | --- | --- | --- |
| Lit-2  (High) | 5 | 2 | 3 | Chromatin assembly complex (CAF-1 cmplex) | 0,4 | 0,4 |
| Lit-9  (High) | 8 | 7 | 7 | LSM 1-7 complex | 0,88 | 1 |
| Lit-5  (High) | 9 | 8 | 12 | RNA polymerase II core complex | 0,89 | 0,67 |
| Lit-11 (High) | 10 | 7 | 10 | Exosome | 0,7 | 0,7 |
| Lit-14 (Low) | 11 | 5 | 5 | TFIID subcomplex | 1 | 1 |
| Lit-4  (Low) | 16 | 4 | 5 | TIP60 HAT complex | 0,25 | 0,8 |

**Ortho CFinder**

| **Modules** | n° of proteins in  predicted module | n° of proteins matched in MIPS complex | **n° of proteine of MIPS complex** | **MIPS complex name** | **P** | **R** |
| --- | --- | --- | --- | --- | --- | --- |
| Ortho-8 (High) | 17 | 4 | 12 | RNA polymerase II core complex | 0,24 | 0,34 |
| Ortho-12 (High) | 11 | 8 | 14 | 20S proteasome | 0,73 | 0,57 |
| Ortho-18 (Medium) | 18 | 13 | 20 | PA700 complex | 0,72 | 0,65 |
| Ortho-7 (Low) | 8 | 4 | 10 | exosome | 0,5 | 0,4 |

**Int CFinder**

| **Modules** | n° of proteins in  predicted module | n° of proteins matched in MIPS complex | n° of proteine of MIPS complex | **MIPS complex name** | **P** | **R** |
| --- | --- | --- | --- | --- | --- | --- |
| Int-2 (High) | 5 | 2 | 3 | Chromatin assembly complex | 0,4 | 0,4 |
| Int-3 (High) | 5 | 1 | 37 | CEN complex | 0,2 | 0,03 |
| Int-4 (High) | 10 |  | na |  |  |  |
| Int-7 (High) | 19 | 5 | 12 | RNA polymerase II core complex | 0,26 | 0,42 |
| Int-18 (Medium) | 20 | 12 | 20 | PA 700 complex | 0,6 | 0,6 |
| Int-13 (Low) | 13 | 5 | 5 | TFIID subcomplex | 0,38 | 1 |

**Literature MCODE**

| **Modules** | n° of proteins in  predicted module | n° of proteins matched in MIPS complex | n° of proteine of MIPS complex | **MIPS complex name** | **P** | **R** |
| --- | --- | --- | --- | --- | --- | --- |
| Lit-4 (High) | 7 | 6 | 10 | Rnase/Mrp complex | 0,86 | 0,6 |
| Lit-11 (Low) | 9 | 1 | 3 | MED18-MED20-MED29 mediator subcomplex | 0,11 | 0,34 |
| Lit-13 (Low) | 21 | 5 | 5 | TFIID subcomplex | 0,24 | 1 |

**Ortho MCODE**

| **Modules** | **n° of proteins in**  **predicted module** | **n° of proteins matched in MIPS complex** | **n° of proteine of MIPS complex** | **MIPS complex name** | **P** | **R** |
| --- | --- | --- | --- | --- | --- | --- |
| Ortho-4 (High) | 13 |  | na |  |  |  |
| Ortho-5 (High) | 9 | 7 | 47 | 60S ribosomal subunit, cytoplasmic | 0,78 | 0,15 |
| Ortho-2 (Low) | 7 | 3 | 5 | TFIID subcomplex | 0,43 | 0,6 |
| Ortho-10 (Low) | 11 | 5 | 5 | Cleavage and polyadenylation factor (CPSF) | 0,46 | 1 |

**Int MCODE**

| **Modules** | **n° of proteins in**  **predicted module** | **n° of proteins matched in MIPS complex** | **n° of proteine of MIPS complex** | **MIPS complex name** | **P** | **R** |
| --- | --- | --- | --- | --- | --- | --- |
| Int-5 (High) | 5 |  | na |  |  |  |
| Int-3  (--) | 13 | 5 | 104 | Nop56p-associated pre-rRNA complex | 0,38 | 0,05 |
| Int-10  (--) | 10 | 6 | 7 | Arp2/3 complex | 0,6 | 0,86 |
| Int-14  (--) | 31 | 7 | 48 | 39S ribosomal subunit, mitochondrial | 0,23 | 0,88 |
