# Supplementary material for: Inferring modules from human protein interactome classes
Source: BMC Syst Biol. 2010 Jul 23;4:102. doi: 10.1186/1752-0509-4-102 (PMC2923113; doi:10.1186/1752-0509-4-102)
Supplement: Additional file 10 — annotation-description. Notes on the computed p-values. [file 1752-0509-4-102-S10.DOC]

The ***GO Term Finder*** attempts to determine whether an observed level of annotation for a group of genes is significant within the context of annotation for all genes within the genome.

With a total population of N genes, in which M have a particular annotation, if we observe x genes with that annotation, in a sample of n genes, then we can calculate the probability of that observation, using the hypergeometric distribution:


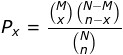


With:

***N***, number of annotated genes from the *gene_association.goa_human* file(*)

***M***, the number of genes with a particular annotation from the *gene_association.goa_human* file (example: GO:0000139: Golgi membrane )

***N***, the number of genes of our module

***x***, the number of genes with ***M*** annotation, in the module of ***n*** genes

Then a ***p-value*** is computed as the probability of seeing ***x*** or more genes with an annotation, out of ***n***, given that ***M*** in the population of ***N*** have that annotation:

***p*_*value* = ∑**

**n**

**j = x**

____________

( ) ( )

( )

M

j

N

n

N-M

n-j

(*) UniProtKB-GOA (GO Annotation@EBI) (<http://www.ebi.ac.uk/GOA/>).

The p-value is corrected with **Bonferroni** and **FDR** (see below).

0.01 is the default **p-value cutoff** used.

**COFECO** (Sun et al, NAR 2009).

COFECO is a web-based tool for a composite annotation of protein complexes with other annotation resources (GO, KEGG, CORUM, Reactome). The processes are implemented as both single and composite enrichment of various combination of annotation resources. In particular for our applications, the annotation was performed using protein complexes, GO and KEGG.
COFECO uses a modified form of the ‘a priori algorithm’, say APA, for composite enrichment that generates sets of associated annotations which co-occur significantly in a set of genes.

The composite enrichment algorithm consists of two processes:

1. generation of composite annotation terms and statistical evaluation of them.
   1. In the generation of composite annotations, APA begins by selecting the set of all single annotation terms that occur in at least *k* concurrent genes.
   2. Then, two terms that occur in at least *k* concurrent genes are merged to a new associated term. The process continues until the longest associated terms are found.
2. In the statistical evaluation process, composite annotations which are significantly enriched in a given gene set are evaluated. As the number of annotation resources increases and *k* decreases, the computational complexity might drastically grow to enumerate all possible compositions of annotation terms. In addition, the protein redundancy among complexes may also lead to huge computation in COFECO analysis.

A greedy algorithm selects the top ranked *K* terms determined by *P*-value calculations at each step of composite annotation. A statistical significance test is applied to all single and associated terms found in the above process, with choice between hypergeometric distribution, binomial test, Fisher's exact test, or chi-squared test (available in COFECO).

The multiple testing correction of *P*-value can be conducted using Bonferroni correction, the Holm–Bonferroni method, or a false discovery rate (FDR) method.

We used default parameters: a cutoff p-value 1, hypergeometric statistical test and FDR p-value correction method.

**Bonferroni** correction is the number of nodes to which the genes of interest are collectively annotated, excluding those nodes that have only a single annotation in the background distribution, which *a priori* cannot be significantly enriched.

In practice, it is a multiple-comparison correction used when several dependent or independent [statistical tests](http://mathworld.wolfram.com/StatisticalTest.html) are being performed simultaneously (while a given [alpha value](http://mathworld.wolfram.com/AlphaValue.html) may be appropriate for each individual comparison, this is not the case for the set of *all* comparisons).

In order to avoid a lot of spurious positives, the [alpha value](http://mathworld.wolfram.com/AlphaValue.html) needs to be lowered to account for the number of comparisons being performed.

Multiple hypothesis correction can be very conservative, as it tries to maintain the probability of getting *any* false positives at a particular alpha level.

**False discovery rate** (**FDR**) control is a [statistical](http://en.wikipedia.org/wiki/Statistics) method used in [multiple hypothesis testing](http://en.wikipedia.org/wiki/Multiple_testing) to correct for [multiple comparisons](http://en.wikipedia.org/wiki/Multiple_comparisons). In a list of rejected hypotheses, FDR controls the [expected](http://en.wikipedia.org/wiki/Expected_value) proportion of incorrectly rejected [null hypotheses](http://en.wikipedia.org/wiki/Null_hypothesis) ([type I](http://en.wikipedia.org/wiki/Type_I_and_type_II_errors) errors).

It is a less conservative procedure for comparison, with greater power than [family-wise error rate](http://en.wikipedia.org/wiki/Familywise_error_rate) (FWER) control, at a cost of increasing the likelihood of obtaining [type I](http://en.wikipedia.org/wiki/Type_I_and_type_II_errors) errors

Typically, a **cut-off** for p-values, known as the alpha level, is chosen, such that p-values below the alpha level are deemed significant.

An **alpha value** is a number
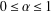
such that P is considered "[significant](http://mathworld.wolfram.com/Significance.html)," where P is a [P-value](http://mathworld.wolfram.com/P-Value.html).
